# Burden of respiratory syncytial virus infection in older and high-risk adults: a systematic review and meta-analysis of the evidence from developed countries

**DOI:** 10.1183/16000617.0105-2022

**Published:** 2022-11-16

**Authors:** Jonathan S. Nguyen-Van-Tam, Maureen O'Leary, Emily T. Martin, Esther Heijnen, Benoit Callendret, Roman Fleischhackl, Christy Comeaux, Thao Mai Phuong Tran, Karin Weber

**Affiliations:** 1 University of Nottingham School of Medicine, Lifespan and Population Health Unit, Nottingham, UK; 2 P95 Epidemiology and Pharmacovigilance, Leuven, Belgium; 3 University of Michigan School of Public Health, Ann Arbor, MI, USA; 4 Janssen Vaccines and Prevention BV, Leiden, The Netherlands; 5 Janssen Research and Development, Beerse, Belgium; 6 Janssen Global Medical Affairs, IDV, Vienna, Austria

## Abstract

**Background:**

Respiratory syncytial virus (RSV) significantly impacts the health of older and high-risk adults (those with comorbidities). We aimed to synthesise the evidence on RSV disease burden and RSV-related healthcare utilisation in both populations.

**Methods:**

We searched Embase and MEDLINE for papers published between 2000 and 2019 reporting the burden and clinical presentation of symptomatic RSV infection and the associated healthcare utilisation in developed countries in adults aged ≥60 years or at high risk. We calculated pooled estimates using random-effects inverse variance-weighted meta-analysis.

**Results:**

103 out of 3429 articles met the inclusion criteria. Among older adults, RSV caused 4.66% (95% CI 3.34–6.48%) of symptomatic respiratory infections in annual studies and 7.80% (95% CI 5.77–10.45%) in seasonal studies; RSV-related case fatality proportion (CFP) was 8.18% (95% CI 5.54–11.94%). Among high-risk adults, RSV caused 7.03% (95% CI 5.18–9.48%) of symptomatic respiratory infections in annual studies, and 7.69% (95% CI 6.23–9.46%) in seasonal studies; CFP was 9.88% (95% CI 6.66–14.43%). Data paucity impaired the calculation of estimates on population incidence, clinical presentation, severe outcomes and healthcare-related utilisation.

**Conclusions:**

Older and high-risk adults frequently experience symptomatic RSV infection, with appreciable mortality; however, detailed data are lacking. Increased surveillance and research are needed to quantify population-based disease burden and facilitate RSV treatments and vaccine development.

## Abstract 2

**In developed countries, older adults and those at risk of complications frequently experience symptomatic respiratory syncytial virus infection. However, more data on key health indicators are required to clarify disease burden and impact in these groups.** https://bit.ly/3zvEHxc

## Introduction

Respiratory syncytial virus (RSV) is a leading cause of acute respiratory tract infection (ARI), including upper (URTI) and lower respiratory tract infection (LRTI). RSV infection is transmitted by direct or indirect contact, with infection rates typically peaking in colder months in temperate climates [1]. Globally, prior to the coronavirus disease 2019 (COVID-19) pandemic, LRTIs represented the fourth cause of overall disability-adjusted life-years at all ages [2], and RSV was the second most common aetiology [3].

The burden of RSV infection is highest in children aged <5 years (global incidence 17.0 (95% uncertainty intervals (UI) 10.6–26.2) per 1000 people) [3, 4], older adults (global incidence 6.3 (95% UI 4.9–7.8) per 1000 people aged >70 years) and adults with underlying comorbidities (such as the immunocompromised and those with an underlying chronic cardiopulmonary disease) [5], who are at risk of severe outcomes of infection (hereafter known as high-risk adults).

Clinical presentation of RSV ranges from a mild cold to a serious respiratory illness with complications comparable to those caused by influenza and other respiratory viruses. These complications can include pneumonia, the need for intensive care unit (ICU) admission [6] and mechanical ventilation, cardiopulmonary complications (in particular exacerbations of congestive heart failure [7] and COPD [8]), and might lead to death [9]. These complications are especially observed in hospitalised RSV patients aged >60 years and in those with underlying health conditions [9].

Currently, there are no specific treatment options for RSV disease among adults, and consequently, several vaccine and therapeutic candidates are under development [10, 11].

To guide this development, robust data on the epidemiology and clinical presentation of RSV infection as well as on associated healthcare utilisation are required. Although research into RSV has increased in recent years, more specific information on the impact of RSV infection in older and high-risk adults is needed [12].

A recently published meta-analysis estimated that in 2015 RSV caused 1.5 million episodes of illness (95% CI 0.3–6.9 million) in adults aged ≥50 years from industrialised countries, of whom an estimated 14.5% (214 000 episodes, 95% CI 100 000–459 000 episodes) were hospitalised and 1.6% (95% CI 0.7–3.8%) died [13]. This meta-analysis provided valuable evidence on disease burden; however, further data on severe outcomes (such as ICU admissions) in older adults as well as on healthcare utilisation are needed. Similarly, overall estimates of the burden of RSV-related disease in high-risk adults are limited.

To strengthen the evidence base on the burden of RSV disease among older and high-risk adults, we conducted a systematic literature review and meta-analysis of 1) the epidemiological burden and clinical presentation of symptomatic RSV infection; 2) the burden of RSV-related severe outcomes and complications of infection; and 3) RSV-related healthcare utilisation. We restricted the study to developed countries as they have comparable healthcare systems.

## Methods

We performed a systematic literature review and meta-analysis in accordance with the Preferred Reporting Items for Systematic Reviews and Meta-analyses guidelines [14].

We prospectively registered the protocol in PROSPERO (registration number CRD42019156730).

### Literature search strategy

We searched Embase (through Ovid) and MEDLINE to identify peer-reviewed articles published between January 2000 and 10 December 2019 using pre-defined terms (supplementary table S1).

### Article selection criteria and data extraction

We included original articles reporting on the burden of symptomatic RSV infection, clinical presentation, symptoms, severe outcomes and RSV-related healthcare utilisation in older adults (aged ≥60 years) and adults aged ≥18 years at risk of complications (high-risk). “High-risk” adults refer to those at risk of complications of infection due to underlying conditions [15, 16] (for a list of conditions selected in this study, see supplementary table S2, definition 1). We defined RSV cases as those with symptomatic laboratory-confirmed infection. RSV clinical outcome definitions are listed in supplementary table S3. We included studies in English and conducted in developed countries recognised as such by the United Nations [17]. Detailed inclusion criteria are compiled in supplementary table S4. One reviewer selected the abstracts using Rayyan [18] and two reviewers screened the full texts and selected the articles that were finally included in the analysis. Any disagreements were resolved by consensus among reviewers. Relevant data from the included articles were extracted using EpiData [19]. Extracted items included the study setting, study period, follow-up time, study population, population age, high-risk group, study design, study outcome, specimen type, testing methodology and the number of study participants. We extracted data on incidence and prevalence of RSV disease burden, clinical presentation (URTI and LRTI), signs and symptoms, severe outcomes (pneumonia, respiratory failure, acute respiratory distress syndrome, cardiopulmonary complications, hospitalisations, ICU admissions, mechanical ventilation and mortality) and healthcare utilisation (outpatient visits, emergency department visits, discharge to care, oxygen therapy and antibiotic use). When possible, we stratified data by age.

### Bias assessment

We used an adapted version of the Newcastle–Ottawa Scale to assess the risk of bias (supplementary table S5A), using a previously described approach [20]. We assessed the study design, study period, representativeness of the study population, case identification, sampling strategy, specimen type, diagnostic assay, outcome assessment and completeness of outcome assessment to categorise the included studies as having either a low or a high risk of bias. Results for each article are displayed in supplementary table S5B and shown in the figures.

### Meta-analysis

We analysed the data and generated summary tables and forest plots using R.3.4.2. [21].

We calculated pooled estimates for outcomes when there were three or more eligible studies; for outcomes with fewer than three studies, data were only presented per study. We reported estimates based on five or more studies as main figures and estimates based on fewer than five studies as supplementary figures or tables. We categorised studies as either annual or seasonal studies, depending on whether data were collected continuously for ≥1 year (annual studies) or whether they were restricted to the respiratory virus season (seasonal studies), and we analysed these separately.

For the older-adult analyses, we stratified data further by population type (community-based or medically attended), by age (≥60 or ≥65 years) and by geographic region (North America, Europe or Western Pacific). We categorised studies as community-based if participants were followed-up prospectively in the community, or as medically attended if data were only collected at points of contact with the health service (including inpatients, outpatients or both).

For the high-risk adult analyses, we stratified data by high-risk subgroup (cardiopulmonary disease, diabetes, chronic kidney disease, immunodeficiency, dementia and functional impairment, as well as institutionalised older adults) and by geographic region. As many studies reported on patients with asthma, an additional specific high-risk subgroup for asthmatic patients was designated. The cardiopulmonary high-risk subgroup does not include the asthmatic subgroup. Patients with HIV, cancer, haematological diseases or immunosuppressive treatment and recipients of transplants were classified as immunodeficient. An overview of included studies is presented in supplementary table S6.

For the analyses of hospitalisation, we excluded populations comprising solely inpatients; we included mixed populations of inpatients and outpatients and we performed an additional sensitivity analysis that included outpatient populations only. There was little difference in the size of the effect estimates generated by the analyses of outpatient populations only; consequently, we report the results of the analyses including both inpatients and outpatients.

We calculated the incidence rate as the number of RSV cases divided by the total person-time followed-up. The population at risk under follow-up was 1) the total number of participants in the cohort, for community-based cohort studies, and 2) the total number of participants in the underlying population, for studies recruiting from medical facilities.

We calculated the proportion as the number of RSV cases divided by either the number of study participants, illness episodes or specimens, depending on the study design. We expressed proportions as percentages.

We used the Wilson “score” method with asymptotic variance without continuity correction to calculate 95% confidence intervals for proportions [22]. To infer the uncertainty for incidence rates, we used the exact 95% confidence interval under the Poisson distribution [23]. We employed random-effects meta-analyses using restricted maximum likelihood to pool information regarding proportions from different studies. For the analyses of incidence rates, we used random-effects models within the maximum likelihood estimation framework. We computed Cochran's Q-test statistics to test for heterogeneity examining the null hypothesis that all studies produce the same effect. We quantified the between-study heterogeneity using the I^2^ statistic, as the power of the Cochran's Q-test is low in the analyses with a small number of studies. This I^2^ statistic quantifies the proportion of total variation in the estimates of treatment effect due to the heterogeneity between studies and is considered to be a better approach for heterogeneity quantification [24].

### Sensitivity analysis for the analysis in high-risk groups

Most data available for high-risk groups came from studies conducted on immunodeficient patients. To study the impact of this patient group on the pooled estimates, we calculated pooled estimates for the high-risk group with and without the immunodeficient group. Some studies reported data for multiple high-risk groups without specifying if these groups were mutually exclusive. In those cases, we first generated pooled estimates including all the observations in a study. We then conducted a sensitivity analysis whereby only a single observation from each study was included. We used a hierarchical approach to select which observations to include using three types of definitions of high-risk groups (supplementary table S2). If a study reported on many high-risk groups, the high-risk group that appears first in the provided list was retained (*e.g.* if the study reported on asthma, diabetes and chronic kidney disease, only asthma data were retained (first order)). The sensitivity analysis demonstrated little change in the effect estimates; therefore, we report the estimates based on the inclusion of all high-risk groups (with the immunodeficient group).

## Results

We identified a total of 3429 articles using our search criteria and included 103 in the review (supplementary figure S1). Of these, 30 studies reported data on older adults, 57 on high-risk groups and 16 on both groups. Most studies were conducted in Europe (50.50%) and North America (38.80%), followed by Australia (7.80%) and Japan (2.90%). More than half of the studies (53.40%) reported data collected continuously over the year (annual data), and 46.60% reported data collected during one or more seasons (seasonal data). Seasonal studies were primarily conducted in winter, except for one study that took place in summer, in Southern California from June to August 2015 [25]. Most studies reported on medically attended populations (77.70%), followed by community-based populations (18.40%) and residents of long-term care facilities (3.90%). Complete descriptions of studies and extracted outcomes are presented in supplementary table S6.

### RSV disease burden and clinical presentation in older adults

Four studies (two on medically attended populations and two in community cohorts) reported on RSV incidence in older adults (aged ≥60 years) (supplementary table S7). The seasonal incidence in older adults was reported by three studies, resulting in the pooled estimate of 16.11 (95% CI 3.52–73.83) cases per 1000 persons per year. These studies showed a very high heterogeneity (I^2^=99.1%, p=0.00). One study reported on the annual RSV incidence in those aged ≥65 years (0.27 (95% CI 0.22–0.33) cases per 1000 persons per year) [26].

**FIGURE 1:**
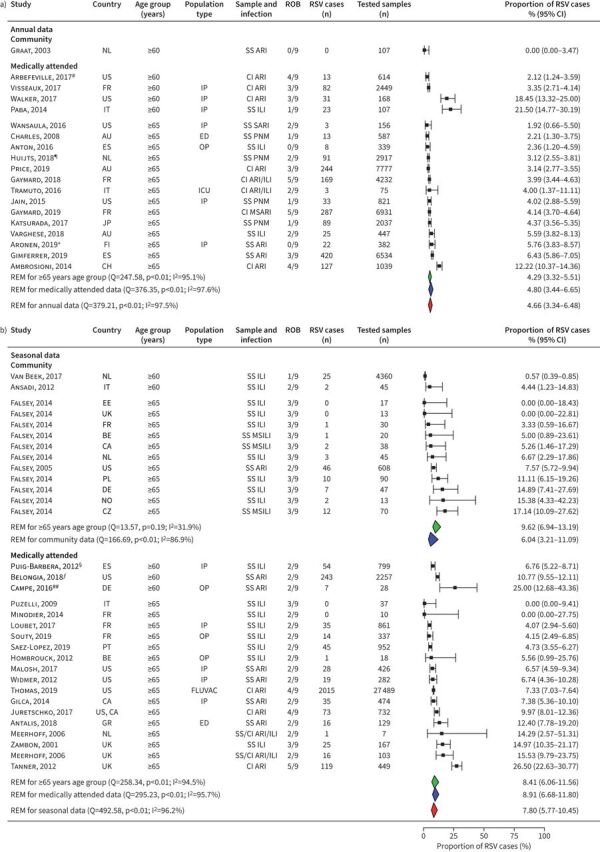
Proportion of respiratory infections attributable to respiratory syncytial virus (RSV) among older adults: a) annual and b) seasonal studies. For each study, the first author, publication year, country of study, participants’ age group, population type, sampling method and respiratory infection, risk-of-bias assessment results (ROB), positive RSV cases, tested samples, proportion of RSV cases (expressed as %) and its 95% confidence interval is given. Estimates stratified by data collection period, population according to the study setting and age are shown. NL: the Netherlands; US: United States of America; FR: France; IT: Italy; AU: Australia; ES: Spain; JP: Japan; FI: Finland; CH: Switzerland; EE: Estonia; UK: United Kingdom; BE: Belgium; CA: Canada; PL: Poland; DE: Germany; NO: Norway; CZ: Czech Republic; PT: Portugal; GR: Greece; REM: random-effect model; Q: Cochran's Q-test; I^2^: I^2^ statistic; SS: systematic sampling; ARI: acute respiratory infection; CI: sampling by clinical indication; IP: inpatients; ILI: influenza-like illness; SARI: severe ARI; ED: emergency department; OP: outpatients; ICU: intensive care unit; PNM: pneumonia; MSARI: moderate to severe ARI; MSILI: moderate to severe ILI; FLUVAC: influenza-vaccinated study population. ^#^: sampling targeted at inpatients with respiratory distress, immunocompromised or critically ill; ^¶^: CAPITA trial, no active community-based follow-up, cases detected from medical facilities; ^+^: very high rates of underlying comorbidities; ^§^: emergency hospitalisations; ^ƒ^: fever and cough included in eligibility criteria most seasons; ^##^: swabbing conducted during influenza season.

41 studies reported on the proportion of RSV infection among older adults with symptomatic respiratory infections, including 31 studies conducted on patients aged ≥65 years (figure 1). According to annual studies (n=18), the proportion of RSV infection ranged from 0.00% to 21.50%, and the pooled estimate reached 4.66% (95% CI 3.34–6.48%). The included studies were highly heterogeneous (I^2^=97.5%, p<0.001), and the estimate was largely driven by studies in medically attended populations and by studies in aged adults ≥65 years. Due to data paucity (n=1) [27], no annual pooled estimate for community-based studies in older adults could be calculated. According to seasonal studies (n=23), the proportion of RSV infection ranged from 0.00% to 26.50%, and the pooled estimate reached 7.80% (95% CI 5.77–10.45%) with high heterogeneity (I^2^=96.2%, p<0.001). When stratified by the study population, the seasonal pooled estimate for medically attended populations was higher than for community cohorts (8.91%, 95% CI 6.68–11.80% and 6.04%, 95% CI 3.21–11.09%, respectively).

We observed little variation in the proportion of RSV-positive cases among older adults when compared by geographic region (supplementary table S8). Among annual studies, the proportion varied from 5.09% (95% CI 3.42–7.50%) in Europe, to 4.49% (95% CI 1.50–12.67%) in North America, to 3.45% (95% CI 2.10–5.61%) in the Western Pacific region. In seasonal studies, the proportion varied from 6.65% (95% CI 4.79–8.87%) in Europe to 6.72% (95% CI 4.78–9.38%) in North America. No seasonal data were available from the Western Pacific region.

Most of the studies in older adults reported data only on symptomatic RSV infection in general and did not report separate data for patients presenting with URTI or LRTI. Consequently, it was not possible to generate specific estimates on URTI or LRTI proportions among older adults. The available data on these outcomes are presented in supplementary table S9. Six studies captured self-reported symptoms associated with RSV infection in older adults, and one recorded data on signs on examination (supplementary table S10). Among older adults, the most frequently self-reported RSV symptoms were cough (with a median of 86.0% patients reporting this symptom across six studies), weakness/malaise (median 86.7%), shortness of breath (median 72.3%), sputum (median 56.1%) and fever (median 53.3%). The most frequently reported sign on examination was wheezing, documented in 20.2% of cases [28].

### RSV infection severe outcomes in older adults

Two community-cohort studies and five studies in medically attended populations reported on RSV severe outcomes in older adults (supplementary table S11A). Overall, an estimated 27.44% (95% CI 18.74–38.29%) of RSV patients developed pneumonia (four studies), 24.48% (95% CI 0.43–96.07%) required hospitalisation (three studies) and 5.01% (95% CI 0.47–37.36%) were admitted to the ICU (three studies). These data should be interpreted with caution because the estimates are based on a limited number of studies combining medially attended and community cohort populations.

**FIGURE 2:**
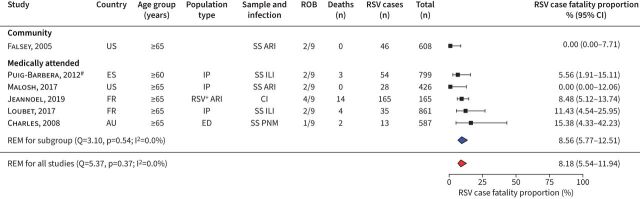
Case fatality proportion among respiratory syncytial virus (RSV)-positive older adults. For each study, the first author, publication year, country of study, participants’ age group, population type, sampling method and respiratory infection, risk-of-bias assessment results (ROB), number of deaths, number of positive RSV cases, total sample size (Total), proportion of deaths among RSV cases (expressed as %) and its 95% confidence interval is given. Estimates stratified by data collection period are shown. US: United States of America; ES: Spain; FR: France; AU: Australia; REM: random-effect model; Q: Cochran's Q-test; I^2^: I^2^ statistic; SS: systematic sampling; ARI: acute respiratory infection; IP: inpatients; ILI: influenza-like illness; CI: sampling by clinical indication; ED: emergency department; PNM: pneumonia. ^#^: emergency hospitalisations.

Using data from five studies in medically attended and one study in community-based older adults, we estimated the overall RSV infection case fatality proportion (CFP) among older adults at 8.18% (95% CI 5.54–11.94%) (figure 2). These studies had low heterogeneity (I^2^=0.0%, p=0.37). There was insufficient data to calculate RSV-related CFPs by geographic region.

### RSV disease burden and clinical presentation in high-risk adults

11 studies (four annual and seven seasonal) reported RSV incidence rates in three high-risk groups (adults aged ≥18 years at risk of complications) (supplementary table S12). Among immunodeficient patients, combining medically attended populations and community cohorts, the annual incidence was 36.88 (95% CI 17.82–76.33) RSV cases per 1000 person-years and the seasonal incidence was seven-fold higher, reaching 260.89 (95% CI 82.33–826.65) RSV cases per 1000 person-years. In patients with cardiopulmonary disease, the seasonal incidence was 19.15 (95% CI 6.06–60.49) RSV cases per 1000 person-years. Estimates in patients with immunodeficiency and cardiopulmonary disease were based on three studies each. A single study reported an incidence of 9.78 (95% CI 3.18–20.04) RSV cases per 1000 person-years in institutionalised older adults [29].

**FIGURE 3:**
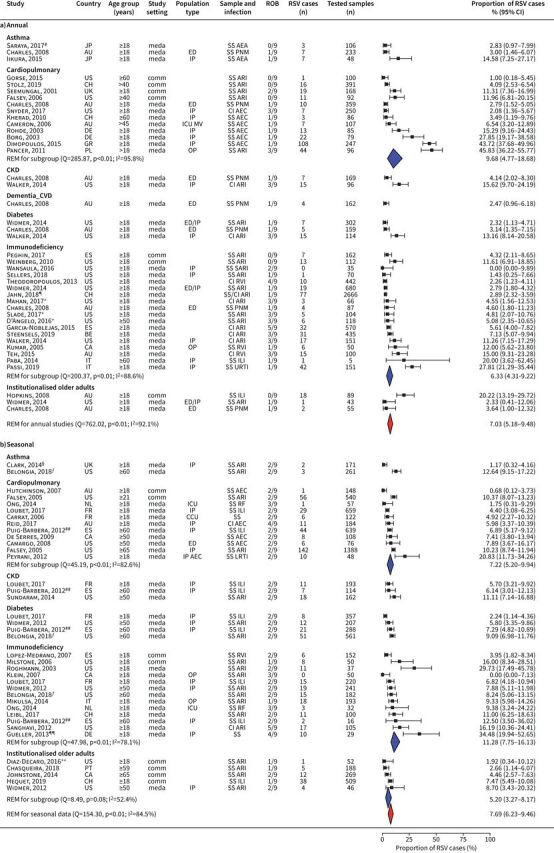
Proportion of respiratory infections attributable to respiratory syncytial virus (RSV) among high-risk adults: a) annual and b) seasonal studies. For each study, the first author, publication year, country of study, participants’ age group, study setting, population type, sampling method and respiratory infection, risk-of-bias assessment results (ROB), positive RSV cases, tested samples, proportion of RSV cases (expressed as %) and its 95% confidence interval is given. Estimates stratified by data collection period and high-risk subgroups are shown. JP: Japan; AU: Australia; US: United States of America; CH: Switzerland; UK: United Kingdom; DE: Germany; GR: Greece; PL: Poland; ES: Spain; BE: Belgium; CA: Canada; IT: Italy; NL: the Netherlands; FR: France; PT: Portugal; REM: random-effect model; Q: Cochran's Q-test; I^2^: I^2^ statistic; meda: medically attended; comm: community-based; SS: systematic sampling; AEA: acute exacerbation of asthma; ED: emergency department; PNM: pneumonia; IP: inpatients; CI: sampling by clinical indication; AEC: acute exacerbation of COPD; ICU MV: intensive care unit, mechanically ventilated; OP: outpatients; ARI: acute respiratory infection; RVI: respiratory virus infection; ILI: influenza-like illness; URTI: upper respiratory tract infection; RF: respiratory failure; CCU: critical care unit; LRTI: lower respiratory tract infection; CKD: chronic kidney disease; CVD: cardiovascular disease. ^#^: excluded COPD, pneumonia, interstitial lung diseases and acute heart failure patients, as well as those with respiratory symptoms due to infections in the past month; ^¶^: included immunocompromised patients with suspicion of infection and/or respiratory symptoms and/or radiologically confirmed lung infiltrates undergoing bronchoscopy; ^+^: patients followed in medical facility; ^§^: inclusion criteria: acute exacerbation of chronic cardiopulmonary illness or acute pulmonary illness (pneumonia, bronchitis, ILI); ^ƒ^: fever and cough included in eligibility criteria most seasons; ^##^: emergency hospitalisations; ^¶¶^: all inpatient haematopoietic stem cell transplant patients enrolled, regardless of whether they had symptoms; ^++^: recruitment during summer.

58 studies assessed the proportion of RSV-positive cases among high-risk groups with respiratory infections. Most of the studies reported on patients with cardiopulmonary disease or immunodeficiency (22 and 21, respectively, out of 58 studies) (figure 3).

According to annual studies (n=32), the proportion of RSV infection among high-risk adults ranged from 0.00% to 45.83%, and the pooled proportion was estimated at 7.03% (95% CI 5.18–9.48%), with high heterogeneity (I^2^=92.1%, p<0.01). A sensitivity analysis excluding immunodeficient patients (definition 2, supplementary table S2) generated a similar estimated proportion of 7.51% (95% CI 4.79–11.60%). Comparing the annual pooled estimates among high-risk subgroups, the RSV proportion was higher among patients with cardiopulmonary disease (9.68%, 95% CI 4.77–18.68%) compared to immunodeficient patients (6.33%, 95% CI 4.31–9.22%). According to seasonal studies (n=26), the proportion of RSV infection among high-risk adults ranged from 0.00% to 34.48% and the pooled proportion was estimated at 7.69% (95% CI 6.23–9.46%). The heterogeneity among seasonal studies was high (I^2^=84.5%, p<0.01). Excluding the immunodeficient patients resulted in an estimated RSV proportion of 6.53% (95% CI 5.24–8.11%). Comparing the seasonal pooled estimates among high-risk subgroups, immunodeficient patients had the highest pooled proportion (11.28%, 95% CI 7.75–16.13%), followed by patients with cardiopulmonary disease (7.22%, 95% CI 5.20–9.94%) and institutionalised older adults (5.20%, 95% CI 3.27–8.17%). As in the annual studies, the immunodeficient group accounted for a large proportion of all seasonal studies.

In annual studies, 11.21% (95% CI 6.45–18.78%) of high-risk adults with symptomatic respiratory infection tested positive for RSV in Europe, 5.44% (95% CI 3.60–8.13%) in North America and 5.32% (95% CI 3.17–8.78%) in the Western Pacific region (supplementary table S8). In seasonal studies, 6.22% (95% CI 4.49–8.55%) of high-risk adults with symptomatic respiratory infection tested positive for RSV in Europe and 10.07% (95% CI 8.05–12.54%) in North America.

56.80% (95% CI 48.13–65.07%) and 44.53% (95% CI 36.83–52.49%) of RSV-positive immunodeficient patients were estimated to have developed URTI and LRTI, respectively (supplementary table S9). Self-reported symptoms affecting >50% of RSV cases included cough, shortness of breath, sputum, nasal congestion, wheezing, discoloured sputum and fever in patients with cardiopulmonary disease (two studies); cough, wheezing and sputum (six studies) among immunodeficient patients; and cough, weakness/malaise and fever (one study) among institutionalised older adults (supplementary table S10). Upon examination, wheezing and crackles were the signs identified in more than half of cardiopulmonary disease patients with RSV infection.

### RSV infection severe outcomes in high-risk adults

Overall, among all high-risk RSV-positive patients, 32.82% (95% CI 23.49–43.74%) required hospitalisation and 26.74% (95% CI 20.40–34.22%) were admitted to the ICU (supplementary table S11B). Among all RSV-positive immunodeficient patients (including community-based and medically attended), 35.33% (95% CI 29.78–41.30%) developed pneumonia (six studies), 20.62% (95% CI 2.22–74.82%) had a respiratory failure (three studies), 24.09% (95% CI 16.35–34.01%) were admitted to the ICU (10 studies), 13.65% (95% CI 7.87–22.63%) required ventilatory support (five studies) and 38.30% (95% CI 29.26–48.23%) were hospitalised (13 studies).

**FIGURE 4:**
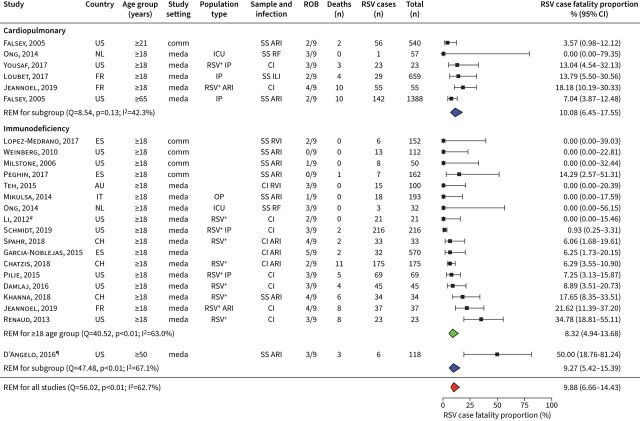
Case fatality proportion among respiratory syncytical virus (RSV)-positive high-risk groups. For each study, the first author, publication year, country of study, participants age group, study setting, population type, sampling method and respiratory infection, risk-of-bias assessment results (ROB), number of deaths, positive RSV cases, total sample size and proportion of deaths among RSV cases (expressed as %) and its 95% confidence interval is given. Estimates stratified by high-risk subgroups are shown. US: United States of America; NL: the Netherlands; FR: France; ES: Spain; AU: Australia; IT: Italy; CH: Switzerland; REM: random-effect model; Q: Cochran's Q-test; I^2^: I^2^ statistic; comm: community-based; meda: medically attended; SS: systematic sampling; ARI: acute respiratory infection; ICU: intensive care unit; RF: respiratory failure; IP: inpatients; CI: sampling by clinical indication; ILI: influenza-like illness; RVI: respiratory viral infection; OP: outpatients. ^#^: patients sampled based on clinical indication, but all RSV positives systematically included in analysis; ^¶^: patients followed in medical facility.

Based on 29 studies (including 18 studies in immunodeficient populations), the estimated RSV infection CFP was 9.88% (95% CI 6.66–14.43%), with substantial heterogeneity observed between studies (I^2^=62.7%; p<0.05), mostly attributable to the heterogeneity observed between some studies from the immunodeficiency high-risk group (figure 4). The CFP in RSV patients with cardiopulmonary disease was estimated at 10.80% (95% CI 6.45–17.55%), and among immunodeficient patients, at 9.27% (95% CI 5.42–15.39%). In Europe, the CFP among RSV-positive high-risk adults was estimated to be 13.00% (95% CI 9.16–18.12%) and, in North America, 7.73% (95% CI 4.18–13.88%) (supplementary table S8). We found insufficient data to calculate RSV-related mortality rates among high-risk adults.

### RSV-related healthcare utilisation in older and high-risk adults

Two studies reported on RSV-related healthcare utilisation in older adults, four in high-risk adults and one in both groups. Among older adults with RSV infection, the studies showed that 76.95–77.91% were treated with antibiotics [5, 28], 13.64–14.81% required oxygen use [28, 30], <1% were discharged to care [28], 0.00–5.35% visited the emergency department [5, 28] and 17.39% were outpatient visitors [5]. Among different high-risk adults with RSV infection, the studies reported that 23.81–50.00% required oxygen use [31–33], 4.17–17.29% were discharged to care [34], 8.93% visited the emergency department [5] and 28.57% were treated as outpatients [5]. We could not calculate pooled estimates for any of the groups due to paucity of data. Data are presented in supplementary table S13.

## Discussion

This systematic literature review and meta-analysis comprehensively synthesised the available evidence on RSV disease burden among older adults aged ≥60 years and high-risk adults in developed countries. Our review was based on 103 articles that included ∼3341 laboratory-confirmed RSV cases. It should be noted throughout that most data were available in high-risk and medically attended populations and that pooled estimates reflect those underlying patient populations more than true community estimates. The results showed a substantial burden of RSV in the adult population, especially those with comorbidities. We estimated that the proportion of RSV cases among respiratory infection was 4.66% in older adults and 7.03% in high-risk adults in annual studies. Severe outcomes were also more frequent among high-risk adults than among older adults. Hospitalisation and ICU admission in high-risk adults were reported in 32.82% and 26.74% of cases, respectively, compared to 24.48% and 5.01% in older adults, and the estimated CFP was 9.88% in high-risk adults and 8.18% in older adults.

In older adults, we estimated a seasonal RSV incidence of 16.11 (95% CI 3.52–73.83) cases per 1000 persons per year (including medically attended and community cohort populations).

As reported by others, RSV incidence is lower among the community cohort (6.7, 95% CI 1.4–31.5 cases per 1000 persons per year for adults aged ≥50 years in industrialised countries [13]) than among hospitalised patients (23.2, 95% CI 11.1–36.8 cases per 1000 persons per year among adults aged ≥65 years [4]).

The annual incidence of RSV in high-risk adults was similar to that recently estimated out of community cohort studies in adults aged ≥18 years with any comorbidity with ARI in industrialised countries (37.6, 95% CI 20.1–70.3 RSV cases per 1000 persons per year) [35]. However, in seasonal studies, we estimated a nine-fold higher incidence, as reported previously by Shi *et al.* [35] (260.89, 95% CI 82.33–826.65 RSV cases per 1000 persons per year compared to 28.4, 95% CI 11.4–70.9 RSV cases per 1000 persons per year, respectively). This difference might be driven by the inclusion of studies on cohorts of immunocompromised patients in our analysis, such as an outpatient cohort of adult bone marrow or peripheral blood stem cell transplant recipients with mild RSV manifestation [36]. Thus, these results should be interpreted with caution.

Our estimate on the proportion of RSV-positive cases causing symptomatic respiratory infections in older adults varied from 4.66% in annual studies to 7.80% in seasonal studies (including community cohort and medically attended). This aligns with previous studies on RSV burden in developed countries where RSV infection was estimated to cause 4.4% (95% CI 3.0–6.5%) of ARI among hospitalised adults aged ≥65 years [13], 10% (95% CI 5–16%) of ARI/influenza-like illness or community-acquired pneumonia among community cohort or medically attended adults aged ≥50 years in Europe and 7% (95% CI 5–9%) in the United States [37], and 12% of ARI among medically attended adults aged ≥50 years without underlying comorbidities in the United States [38]. Two recent studies in those aged >60 years support these estimates, reporting that 5.4% of hospitalised patients with acute respiratory illnesses [9] and 5.6% of community-dwelling adults [39] are RSV-infected. Altogether, the proportion of respiratory infection caused by RSV among older adults ranged from 4% to 12%.

The proportion of RSV-positive cases among high-risk groups with respiratory infection was similar to that previously estimated by two meta-analyses [37, 38]. Those studies demonstrated that RSV infection accounted for 8.6% to 20.0% of all respiratory viral infections among immunocompromised patients [37] and 8% to 13% of infections among adults with chronic cardiopulmonary diseases who were hospitalised during the winter season [38].

Cough was the most frequently reported symptom followed by shortness of breath, sputum and fever, consistent with previous studies in older and high-risk adults [40]. Severe outcomes due to RSV infection (pneumonia, hospitalisation, ICU admission and death) were more frequent among high-risk patients, mostly immunodeficient, than among older adults. Pneumonia was the most frequent complication in older adults and immunodeficient patients (27.44% and 35.33, respectively). Even higher estimates were recently reported in the study by Tseng *et al*. [9], in which it was estimated that >65% of RSV cases of hospitalised patients aged >60 years (with and without comorbidities) developed pneumonia, probably reflecting underlying frailty and age. After pneumonia, hospitalisation was the second most frequent severe outcome, affecting 24.48% of older adults and 32.82% of high-risk adults. ICU admission was required five times more often among high-risk patients than among older adults. According to our pooled estimate from 11 studies (mostly on immunodeficient patients), more than a quarter of high-risk patients required ICU admission [9]. This indicates the higher risk of severe disease and poor outcome in high-risk patients, especially among immunodeficient patients.

The CFP among older adults mostly hospitalised reached 8.18% (95% CI 5.54–11.94%). The CFP was 10.80% (95% CI 6.45–17.55%) among cardiopulmonary patients and 9.27% (95% CI 5.42–15.39%) among immunodeficient adults, demonstrating similar results to those recently estimated for mostly immunocompetent RSV-ARI high-risk adults (11.7%, 95% CI 5.8–23.4%) [35]. According to another study, cumulative mortality consistently increases after hospitalisation, reaching 25.8% in adults aged ≥60 years after 1 year of admission [9]. The high CFP among immunocompetent older adults and those with comorbidities suggests that vaccination of at-risk patients could be a useful intervention to prevent RSV-related mortality.

Although RSV is the second most common aetiology of LRTI [3] after pneumococcal pneumonia, and it might lead to severe complications, our systematic literature review identified a paucity of data on disease burden and healthcare utilisation. This limited our ability to estimate the incidence or prevalence of 1) different clinical presentations of RSV disease; 2) the RSV-related complications; and 3) the healthcare utilisation such as duration of hospitalisation. Data stratification by narrower age groups could not be investigated either due to the lack of data. It has been reported that RSV might have a major impact on hospitalisation and worse outcomes among the elderly and patients with underlying comorbidities compared to influenza [41–43]. Whereas influenza is subject to well-resourced seasonal surveillance and control strategies, including targeted vaccination programmes and treatment, this is not the case for RSV. Improved surveillance, including the adoption of the standardised case definitions, and criteria for testing and reporting, similarly to that seen for influenza, would enable the generation of more robust estimates of the burden of disease and the identification of high-risk patients that needs special management and treatment. This could be achieved in the future as multiplex testing is now more widely adopted in response to the severe acute respiratory syndrome coronavirus 2 (SARS-CoV-2) pandemic.

A good surveillance system is especially important, since RSV circulation has been altered due to the COVID-19 pandemic. During 2020–2021, RSV season was either completely missed (Brazil, Chile, Japan, Canada and South Korea) or delayed by an average of 39 weeks due to the effect of the nonpharmacological contingency interventions (social isolation, movement and gathering restriction, school and workplace closing, face-mask policies) [44–47]. The virus resurged after restrictions were lifted and schools reopened [48]. According to the largest study that examined clinical outcomes of co-infection with influenza viruses, RSV or adenoviruses in 6965 adults with SARS-CoV-2 in the United Kingdom, RSV–COVID-19 dual infection affected 3.165% (220 out of 6965) of the patients with COVID-19, and was not associated with increased odds of receiving invasive mechanical ventilation, as was reported for influenza virus or adenovirus coinfection [49].

Our study is strengthened by the application of strict inclusion criteria, the assessment of the risk of bias, and the use of sensitivity and stratified analyses to thoroughly interrogate the available data. However, our study had several limitations. First, the included papers used different methods, and this might affect the comparability of the studies. Second, the true burden of RSV might be underestimated due to inclusion of only laboratory-confirmed cases. Third, medically attended and inpatient populations predominate, which potentially leads to underestimating the true RSV disease burden.

This study strengthens the evidence base for the impact of RSV in older adults, and especially in adults with comorbidities. Comorbidities in older RSV patients are common, particularly chronic pulmonary and chronic cardiac conditions [50]. The combination of older age and underlying comorbidities may further increase the risk of severe outcomes of infection. Considering the increasingly ageing population in high-income countries [51] and the high proportion of adults aged ≥60 years with comorbidities (estimated at 22–31% of the world's population when considering diseases causing an increased risk of severe COVID-19 [52]), the population at risk of RSV severe outcomes is substantial, and RSV intervention should be prioritised. To further characterise the RSV disease burden and to facilitate the development of RSV vaccines and treatments, increased RSV surveillance and understanding of key RSV epidemiological indicators, healthcare utilisation, risk factors and severe outcomes across risk groups and older adults is needed.

## Supplementary material

10.1183/16000617.0105-2022.Supp1 **Please note:** supplementary material is not edited by the Editorial Office, and is uploaded as it has been supplied by the author.
Supplementary material ERR-0105-2022.SUPPLEMENT
