# Supplementary material for: Burden of respiratory syncytial virus infection in older and high-risk adults: a systematic review and meta-analysis of the evidence from developed countries
Source: Eur Respir Rev. 2022 Nov 16;31(166):220105. doi: 10.1183/16000617.0105-2022 (PMC9724807; doi:10.1183/16000617.0105-2022)
Supplement: Supplementary file 1 [file ERR-0105-2022.SUPPLEMENT.pdf]

**Title**

Burden of respiratory syncytial virus infection in older and high-risk adults: a systematic review and meta-analysis of the evidence from developed countries

## SUPPLEMENTARY FIGURE LEGEND

**Supplementary Figure 1.** PRISMA flow diagram.

## SUPPLEMENTARY TABLES LEGEND

**Supplementary Table 1**. Embase and Medline search terms.
**Supplementary Table 2.** HR groups definitions and hierarchy for retention of data from studies reporting data in multiple HR groups in overall pooled analyses.
**Supplementary Table 3**. Clinical outcome definitions of RSV infection.
**Supplementary Table 4**. Inclusion and exclusion criteria.
**Supplementary Table 5.** ROB assessment tool **(A.)** and ROB assessment results per included study **(B.).**
**Supplementary Table 6**. Characteristics of included studies.
**Supplementary Table 7.** RSV incidence in older adults (annual and seasonal studies).
**Supplementary Table 8**. Estimated proportion of symptomatic respiratory infection attributable to RSV and estimated RSV case fatality proportion among older adults and HR adults by geographical location.
**Supplementary Table 9**. URTI, LRTI and bronchitis proportion among older adults and HR groups.
**Supplementary Table 10**. RSV signs and symptoms description in older and high-risk adults.
**Supplementary Table 11.** RSV severe outcomes in older adults **(A.)** and HR adults **(B.).**
**Supplementary Table 12**. RSV incidence in HR adults (annual and seasonal studies).
**Supplementary Table 13**. RSV related healthcare utilisation of older and HR adults.

## SUPPLMENTARY FIGURE

**Supplementary Figure 1.** PRISMA flow diagram.

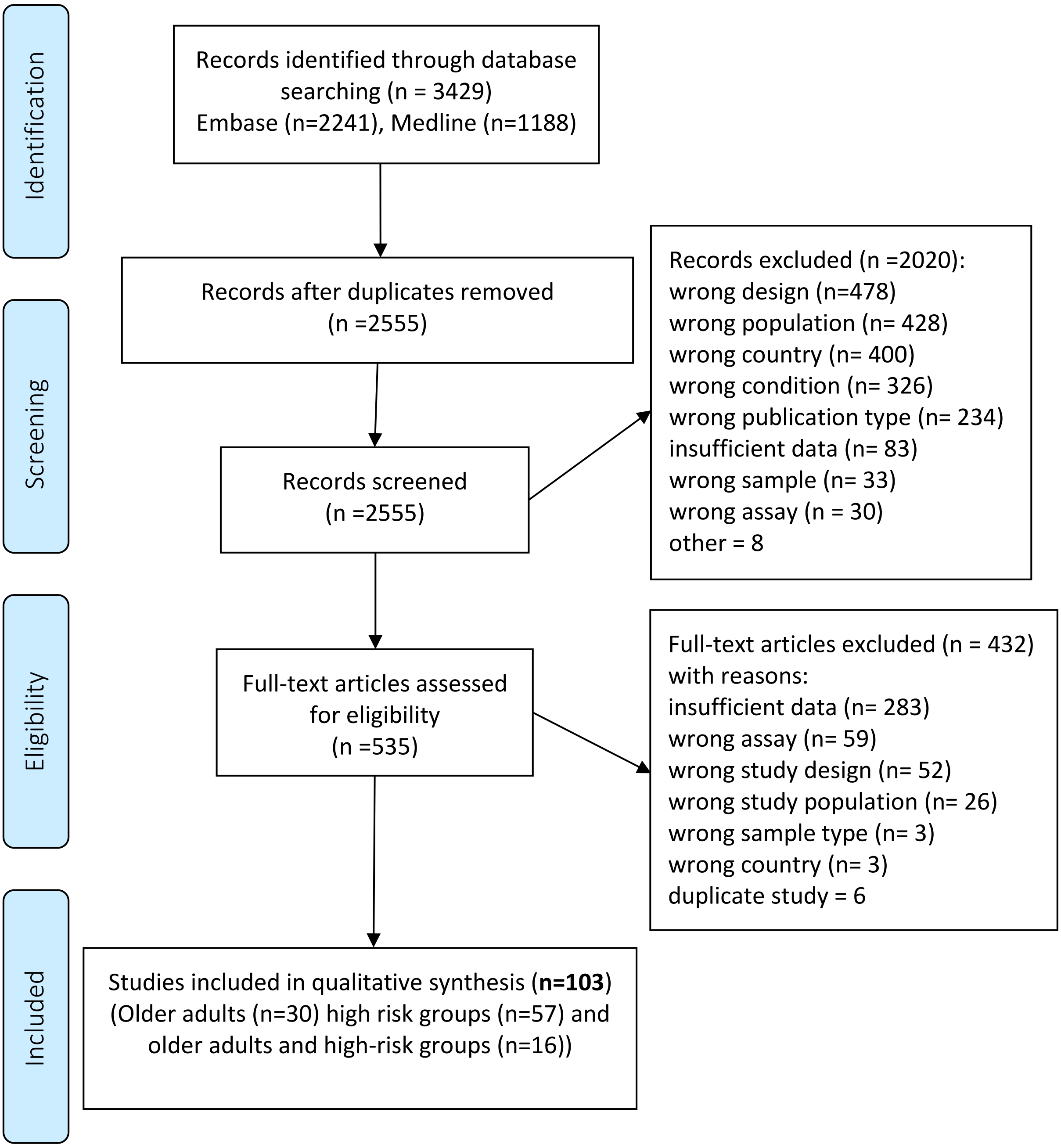

## SUPPLEMENTARY TABLE

**Supplementary Table 1.** Embase and Medline search terms.

| **Embase search terms (Ovid); 2241 articles** | |
|---|---|
| 1 | exp Human respiratory syncytial virus/ or exp respiratory syncytial virus infection/ or rsv.mp. or exp Respiratory syncytial pneumovirus/ or exp respiratory syncytial virus vaccine/ |
| 2 | respiratory syncytial virus.mp. |
| 3 | 1 or 2 |
| 4 | respiratory tract infection.mp. or exp respiratory tract infection/ |
| 5 | ARI.mp. |
| 6 | upper respiratory tract infection.mp. or exp upper respiratory tract infection/ |
| 7 | URTI.mp. |
| 8 | lower respiratory tract infection.mp. or exp lower respiratory tract infection/ |
| 9 | LRTI.mp. |
| 10 | ALRTI.mp. |
| 11 | ALRI.mp. |
| 12 | bronchitis.mp. or exp bronchitis/ |
| 13 | cough.mp. or exp coughing/ |
| 14 | exp sputum/ or sputum.mp. |
| 15 | haemoptysis.mp. or exp hemoptysis/ |
| 16 | hemoptysis.mp. |
| 17 | wheezing.mp. or exp wheezing/ |
| 18 | shortness of breath.mp. or exp dyspnea/ |
| 19 | dyspnea.mp. |
| 20 | runny nose.mp. or exp rhinorrhea/ |
| 21 | rhinorrhea.mp. |
| 22 | rhinorrhoea.mp. |
| 23 | congested nose.mp. |
| 24 | blocked nose.mp. |
| 25 | sore throat.mp. or exp sore throat/ |
| 26 | pharyngitis.mp. or exp pharyngitis/ or exp viral pharyngitis/ |
| 27 | headache.mp. or exp headache/ |
| 28 | exp fatigue/ or fatigue.mp. |
| 29 | exp fever/ or fever.mp. |
| 30 | pyrexia.mp. |
| 31 | exp myalgia/ or myalgia.mp. |
| 32 | muscle pain.mp. |
| 33 | exp respiratory distress/ or respiratory compromise.mp. |
| 34 | difficulty breathing.mp. |
| 35 | weakness.mp. or exp weakness/ |
| 36 | malaise.mp. or exp malaise/ |
| 37 | respiratory distress.mp. or exp respiratory distress/ |
| 38 | tachypnoea.mp. or exp tachypnea/ |
| 39 | tachypnea.mp. |
| 40 | reduced breath sounds.mp. or exp abnormal respiratory sound/ |
| 41 | crackles.mp. or exp crackle/ |
| 42 | rales.mp. |
| 43 | rhonchi.mp. |
| 44 | oxygen saturation.mp. or exp oxygen saturation/ |
| 45 | pneumonia.mp. or exp pneumonia/ |
| 46 | respiratory failure.mp. or exp respiratory failure/ |
| 47 | hypoxia.mp. or exp hypoxia/ |
| 48 | hypoxic.mp. |

| **Embase search terms (Ovid); 2241 articles** | |
|---|---|
| 49 | hypercapnia/ or hypercapnic.mp. |
| 50 | hypercapnia.mp. |
| 51 | hypercapnoea.mp. |
| 52 | hypercapnea.mp. |
| 53 | adult respiratory distress syndrome.mp. or exp adult respiratory distress syndrome/ |
| 54 | ARDS.mp. |
| 55 | cardiorespiratory failure.mp. or exp cardiopulmonary insufficiency/ |
| 56 | cardiopulmonary complications.mp. |
| 57 | arrhythmia.mp. or exp heart arrhythmia/ |
| 58 | congestive heart failure.mp. or exp congestive heart failure/ |
| 59 | myocardial infarction.mp. or exp heart infarction/ |
| 60 | stroke.mp. or cerebrovascular accident/ |
| 61 | chronic obstructive pulmonary disease.mp. or exp chronic obstructive lung disease/ |
| 62 | copd.mp. |
| 63 | chronic obstructive lung disease.mp. |
| 64 | exp asthma/ or asthma.mp. |
| 65 | exp disease exacerbation/ or exacerbation .mp. |
| 66 | exp complication/ or complication .mp. |
| 67 | exp hospital readmission/ or hospital .mp. or exp hospital admission/ |
| 68 | intensive care.mp. or exp intensive care/ |
| 69 | ICU.mp. or exp intensive care unit/ |
| 70 | high dependency.mp. or exp high dependency unit/ |
| 71 | emergency care.mp. or exp emergency care/ |
| 72 | mechanical ventilation.mp. or exp artificial ventilation/ |
| 73 | exp mortality/ or mortality.mp. or exp mortality rate/ |
| 74 | exp death/ or death.mp. |
| 75 | case fatality.mp. |
| 76 | exp outpatient care/ or outpatient.mp. or exp outpatient/ or exp outpatient department/ |
| 77 | primary care.mp. or exp primary medical care/ |
| 78 | general practice.mp. or exp general practice/ |
| 79 | emergency department.mp. or exp emergency ward/ |
| 80 | exp emergency health service/ or A&E.mp. |
| 81 | exp rehabilitation/ or rehab .mp. |
| 82 | nursing.mp. or exp nursing home/ |
| 83 | long-term care.mp. or exp long term care/ |
| 84 | exp home oxygen therapy/ or exp oxygen therapy/ or oxygen.mp. or exp oxygen/ |
| 85 | antibiotic.mp. or exp antibiotic agent/ |
| 86 | antimicrobial.mp. |
| 87 | exp incidence/ or incidence.mp. |
| 88 | prevalence.mp. or exp prevalence/ |
| 89 | morbidity.mp. or exp morbidity/ |
| 90 | burden.mp. or global disease burden/ or disease burden/ |
| 91 | utilisation.mp. or exp health care utilisation/ |
| 92 | 4 or 5 or 6 or 7 or 8 or 9 or 10 or 11 or 12 or 13 or 14 or 15 or 16 or 17 or 18 or 19 or 20 or 21 or 22 or 23 or 24 or 25 or 26 or 27 or 28 or 29 or 30 or 31 or 32 or 33 or 34 or 35 or 36 or 37 or 38 or 39 or 40 or 41 or 42 or 43 or 44 or 45 or 46 or 47 or 48 or 49 or 50 or 51 or 52 or 53 or 54 or 55 or 56 or 57 or 58 or 59 or 60 or 61 or 62 or 63 or 64 or 65 or 66 or 67 or 68 or 69 or 70 or 71 or 72 or 73 or 74 or 75 or 76 or 77 or 78 or 79 or 80 or 81 or 82 or 83 or 84 or 85 or 86 or 87 or 88 or 89 or 90 or 91 |
| 93 | 3 and 92 |
| 94 | limit 93 to (human and english and yr="2000 -Current" and (adult <18 to 64 years> or aged >65 years>)) |

| **Medline search terms (Ovid); 1188 articles** | |
|---|---|
| 1 | respiratory syncytial virus.mp. or exp Respiratory Syncytial Viruses/ |
| 2 | exp Respiratory Syncytial Virus Infections/ or RSV.mp. |
| 3 | 1 or 2 |
| 4 | respiratory tract infection.mp. or exp Respiratory Tract Infections/ |
| 5 | acute respiratory tract infection.mp. |
| 6 | ARI.mp. |
| 7 | upper respiratory tract infection.mp. |
| 8 | URTI.mp. |
| 9 | lower respiratory tract infection.mp. |
| 10 | LRTI.mp. |
| 11 | bronchitis.mp. |
| 12 | respiratory tract disease.mp. or exp Respiratory Tract Diseases/ |
| 13 | exp Cough/ or cough.mp. |
| 14 | sputum.mp. or exp Sputum/ |
| 15 | exp Hemoptysis/ or haemoptysis.mp. |
| 16 | hemoptysis.mp. |
| 17 | wheezing.mp. or exp Respiratory Sounds/ |
| 18 | shortness of breath.mp. or exp Dyspnea/ |
| 19 | dyspnea.mp. |
| 20 | exp Rhinitis/ or rhinorrhea.mp. |
| 21 | rhinorrhoea.mp. |
| 22 | nasal congestion.mp. |
| 23 | blocked nose.mp. |
| 24 | sore throat.mp. or exp Pharyngitis/ |
| 25 | pharyngitis.mp. |
| 26 | headache.mp. or exp Headache/ |
| 27 | exp Fatigue/ or fatigue.mp. |
| 28 | fever.mp. or exp Fever/ |
| 29 | pyrexia.mp. |
| 30 | myalgia.mp. or exp Myalgia/ |
| 31 | muscle pain.mp. |
| 32 | exp Respiratory Insufficiency/ or respiratory compromise.mp. |
| 33 | weakness.mp. |
| 34 | malaise.mp. |
| 35 | exp Tachypnea/ or tachypnea.mp. |
| 36 | tachypnoea.mp. |
| 37 | reduced breath sounds.mp. |
| 38 | crackles.mp. or exp Respiratory Sounds/ |
| 39 | rales.mp. |
| 40 | rhonchi.mp. |
| 41 | oxygen saturation.mp. |
| 42 | exp Pneumonia/ or pneumonia.mp. |
| 43 | respiratory failure.mp. |
| 44 | hypoxia.mp. or exp Hypoxia/ |
| 45 | hypoxic.mp. |
| 46 | exp Hypercapnia/ or hypercapn .mp. |
| 47 | exp Respiratory Distress Syndrome, Adult/ or respiratory distress.mp. |
| 48 | ARDS.mp. |
| 49 | cardiorespiratory failure.mp. |
| 50 | cardiopulmonary insufficiency.mp. |
| 51 | cardiopulmonary complications.mp. |
| 52 | arrhythmia.mp. or exp Arrhythmias, Cardiac/ |
| 53 | congestive heart failure.mp. or exp Heart Failure/ |

| **Medline search terms (Ovid); 1188 articles** | |
|---|---|
| 54 | myocardial infarction.mp. or exp Myocardial Infarction/ |
| 55 | stroke.mp. or exp Stroke/ |
| 56 | cerebrovascular accident.mp. |
| 57 | COPD.mp. or exp Pulmonary Disease, Chronic Obstructive/ |
| 58 | chronic obstructive pulmonary disease.mp. |
| 59 | asthma.mp. or exp Asthma/ |
| 60 | exacerbation.mp. |
| 61 | complicatio .mp. |
| 62 | hospital admission.mp. or exp Hospitalisation/ |
| 63 | hospital .mp. |
| 64 | exp Intensive Care Units/ or exp Critical Care/ or ICU.mp. or exp Critical Illness/ |
| 65 | intensive care.mp. |
| 66 | high dependency.mp. |
| 67 | emergency care.mp. or exp Emergency Medical Services/ |
| 68 | mechanical ventilation.mp. or exp Respiration, Artificial/ |
| 69 | artificial respiration.mp. |
| 70 | exp Mortality/ or mortality.mp. |
| 71 | death.mp. or exp Death/ |
| 72 | case fatality.mp. |
| 73 | outpatient.mp. or exp Outpatients/ |
| 74 | primary care.mp. or exp Primary Health Care/ |
| 75 | general practice.mp. or exp General Practice/ |
| 76 | A&E.mp. |
| 77 | emergency.mp. |
| 78 | emergency department.mp. or exp Emergency Service, Hospital/ |
| 79 | exp Rehabilitation/ or exp Rehabilitation Centers/ or rehab .mp. |
| 80 | nursing care.mp. or exp Nursing Care/ |
| 81 | long term care.mp. or exp Long-Term Care/ |
| 82 | exp Oxygen Inhalation Therapy/ or oxygen therapy.mp. or exp Oxygen/ |
| 83 | antibiotic.mp. or exp Anti-Bacterial Agents/ |
| 84 | exp Drug Prescriptions/ or exp Prescription Drugs/ or prescri .mp. or exp Prescriptions/ |
| 85 | antimicrobial.mp. |
| 86 | drug utilisation.mp. or exp Drug Utilisation/ |
| 87 | morbidity.mp. or exp Morbidity/ |
| 88 | incidence.mp. |
| 89 | prevalence.mp. |
| 90 | burden of disease.mp. |
| 91 | utili#ation.mp. |
| 92 | 4 or 5 or 6 or 7 or 8 or 9 or 10 or 11 or 12 or 13 or 14 or 15 or 16 or 17 or 18 or 19 or 20 or 21 or 22 or 23 or 24 or 25 or 26 or 27 or 28 or 29 or 30 or 31 or 32 or 33 or 34 or 35 or 36 or 37 or 38 or 39 or 40 or 41 or 42 or 43 or 44 or 45 or 46 or 47 or 48 or 49 or 50 or 51 or 52 or 53 or 54 or 55 or 56 or 57 or 58 or 59 or 60 or 61 or 62 or 63 or 64 or 65 or 66 or 67 or 68 or 69 or 70 or 71 or 72 or 73 or 74 or 75 or 76 or 77 or 78 or 79 or 80 or 81 or 82 or 83 or 84 or 85 or 86 or 87 or 88 or 89 or 90 or 91 |
| 93 | 3 and 92 |
| 94 | limit 93 to (english language and humans and yr="2000 -Current" and "all adult (19 plus years)") |

**Supplementary Table 2.** High risk groups definitions and hierarchy for retention of data from studies reporting data in multiple HR groups in overall pooled analyses.

| High risk group definition | Includes the following underlying conditions: |
|---|---|
| Definition 1 | - cardiopulmonary disease (chronic obstructive pulmonary disease (COPD), chronic heart disease (coronary heart failure, coronary artery disease (e.g., angina pectoris, ischemic cardiomyopathy, history of myocardial infarct, history of coronary artery bypass graft or coronary artery stent)<br>- asthma<br>- diabetes<br>- chronic kidney disease (CKD)<br>- immunodeficiency or immunosuppressive diseases (severe combined immunodeficiency, leukaemia or hematopoietic cell or lung transplant)<br>- dementia or stroke<br>- institutionalized older adults<br>- functional impairment |
| Definition 2 | - As HR group definition 1 but excluding patients with immunodeficiency or immunosuppressive diseases (severe combined immunodeficiency, leukaemia or hematopoietic cell or lung transplant). |
| Definition 3 | - cardiopulmonary disease (chronic obstructive pulmonary disease (COPD), chronic heart disease (coronary heart failure, coronary artery disease (e.g., angina pectoris, ischemic cardiomyopathy, history of myocardial infarct, history of coronary artery bypass graft or coronary artery stent))<br>- asthma |
| **Hierarchy among HR group** | **Rationale** |
| 1. Asthma<br>2. Cardiopulmonary | Risk groups included in the most specific definition of HR (definition 3). Asthma prioritised for inclusion as data for this group were less frequently reported |
| 3. Diabetes<br>4. CKD<br>5. Dementia<br>6. Institutionalised older adults | Groups included in the next most specific definition (definition 2). Ordered according to their prevalence in the general population* |
| 7. Immunodeficient | Only included in definition 1, the least specific definition of HR groups. |

** 1 in 11 adults worldwide aged 20-79 have diabetes (diabetes atlas https://diabetesatlas.org/en/); Global prevalence of CKD = 9.1%[1]. 5-8% of global population have dementia (WHO https://www.who.int/news-room/fact-sheets/detail/dementia); 3.1% of US[2] and 3.2%of UK[3] adults aged 65+ reside in long term care homes*

**Supplementary Table 3.** Clinical outcome definitions of RSV infection.

| Outcome | Outcome definition |
|---|---|
| Symptomatic RSV infection | Based on clinical diagnosis |
| Upper respiratory tract infection (URTI) | Based on clinical diagnosis |
| Lower respiratory tract infection (LRTI) | Based on clinical diagnosis |
| Bronchitis | Based on clinical diagnosis |
| Symptoms & signs | Fever will be based on patient self-report and for the assessment of signs fever will be defined as a measured temperature of ≥380C for those aged 18-59 years and ≥37.50C for those aged ≥60 years |
| Pneumonia | Based on clinical diagnosis |
| Respiratory failure | Respiratory failure will refer to acute respiratory failure and will include hypoxic (type 1) and hypercapnic (type 2) respiratory failure, as well as unspecified types of acute respiratory failure. Respiratory failure can be based on the definitions used in the source paper. The definition used will be noted in the data extraction form, to aid data interpretation and inform possible additional analyses. |
| Acute respiratory distress syndrome (ARDS) | ARDS will not be subject to any particular definition but will be based on the definitions used in the source papers; although similar to respiratory failure, the definition used will be noted in the data extraction form. |
| Cardiopulmonary complications | Cardiopulmonary complications will include lower respiratory tract complications (pneumonia and exacerbations of COPD or asthma) and cardiovascular complications (arrhythmia, congestive heart failure exacerbation, myocardial infarction and stroke), in accordance with the definition used by Volling et al.[4]. |

**Supplementary Table 4.** Inclusion and exclusion criteria.

| Parameter | Criteria |
|---|---|
| Population | 1. The general population of adults aged 60 years and older.<br>2. High risk adults aged 18 years and older (Definition 1, Supplementary Table2) |
| Outcomes | 1. Incidence and proportion of symptomatic RSV infection<br>2. RSV-related URTI, LRTI, bronchitis<br>3. RSV-related signs and symptoms<br>4. RSV-related CAP, respiratory failure, ARDS, cardiopulmonary complications, hospitalisations, ICU admissions, mechanical ventilation and case fatality rate.<br>5. RSV related outpatient and emergency department consultations and discharges to skilled nursing care, oxygen therapy and antibiotic use |
| Time | 6. Studies dating from 2000 to 10.12.2020 |
| Study design | Included:<br>− Peer reviewed observational studies on RSV infection, signs and symptoms, outcomes of infection, and healthcare utilisation including cohort, case-control and surveillance reports.<br>− Modelling studies (if data can be extracted on the underlying estimates of RSV burden used to generate the model).<br>− Conference abstracts (if both numerator and denominator data can be extracted on the study outcomes).<br>− Interventional trials (if epidemiological data can be extracted from a control group).<br>− Studies of point of care testing (if PCR test data from a clearly defined unbiased sample were conducted).<br>− Validation studies of laboratory assays based on systematic testing or testing of an unbiased sample of routinely received specimens.<br>− Longitudinal studies of HR groups (such as stem-cell transplant patients), including studies of pharmaceutical interventions such as the use of palivizumab (if the follow-up time is clearly defined and can be used to generate an estimate in person-time).<br>− Longitudinal and surveillance studies in long-term care facilities, reporting annual or seasonal incidence of RSV (as opposed to reporting the attack rate during an outbreak).<br><br>Excluded:<br>− Literature review articles (reference lists will be scanned to identify relevant articles).<br>− Outbreak reports, including reports of outbreaks in long-term care facilities.<br>− Reports on hospital-acquired infections.<br>− Case reports.<br>− In vitro studies.<br>− Animal studies.<br>− Immunogenicity, safety and human challenge studies.<br>− Environmental studies.<br>− Economic and quality of life studies.<br>− Knowledge, attitude and perception studies.<br>− Studies in travelers & children.<br>− Studies lacking clearly defined denominator data (such as data on the follow-up time).<br>− Studies lacking clearly defined study populations.<br>Studies not reporting data in those aged ≥60 (general population) or in HR groups aged ≥18 (for instance if data are only reported for all adults over 18 years of age and separate estimates for those aged 60 and over or HR groups are not reported) |

| **Parameter** | **Criteria** |
|---|---|
| | |
| Other | Included:<br>– Studies from developed countries[5] including: Austria, Belgium, Denmark, Finland, France, Germany, Greece, Ireland, Italy, Luxembourg, Netherlands, Portugal, Spain, Sweden, UK, Bulgaria, Croatia, Cyprus, Czech Republic, Estonia, Hungary, Latvia, Lithuania, Malta, Poland, Romania, Slovakia, Slovenia, Iceland, Norway, Switzerland, Australia, Canada, Japan, New Zealand, USA.<br>– English language articles.<br><br>RT-PCR confirmed RSV cases only (including both rapid molecular tests and multiplex molecular tests of respiratory specimens including nose and throat swabs, nose swabs, oropharyngeal/throat swabs, nasal washes, nasopharyngeal swabs and aspirates, sputum, bronchoscopy and bronchoalveolar lavage samples).<br>Excluded:<br>– RSV cases identified through viral culture, rapid antigen detection tests, direct fluorescent antibody tests.<br>– Studies of asymptomatic RSV infection. |

**Supplementary Table 5.A.** ROB assessment tool.

| Domain | Category | Risk of Bias |
|---|---|---|
| 1. Study design | Prospective enrolment of cases | Low |
| | Other studies | High |
| 2. Study period | At least one complete year | Low |
| | Less than a complete year, for instance:<br>One or more influenza seasons | High |
| 3. Representativeness of study population | Largely representative of target population | Low |
| | Selection of particular groups of participants that might bias estimates including:<br>1. ILI cases.<br>2. Hospitalised persons.<br>3. Vaccinated persons.<br>4. Other selection criteria that might affect estimates.<br>5. Selection of participants not described. | High |
| 4. Case identification | Cases identified using standardised case definition & approach | Low |
| | No standardised case definition or process for case identification, or process not described | High |
| 5. Sampling strategy | ≥90% of eligible cases tested or a systematic sample of cases tested | Low |
| | <90% of eligible cases tested, or non-systematic sampling strategy used, or sampling strategy not described, or proportion of eligible cases tested not reported | High |
| 6. Specimen type | Any of nose & throat swab, nose swab, nasal wash, sputum, nasopharyngeal swab or aspirate, bronchoscopy, bronchoalveolar lavage +/- throat swab | Low |
| | 100% throat swabs | High |
| 6. Diagnostic test | 100% PCR testing | Low |
| | Mix of diagnostic assays including PCR, immunofluorescence, viral culture, antibody tests | High |
| 7. Outcome assessment | Outcomes identified using standardised approach including:<br>1. Clinical assessment.<br>2. Medical record review.<br>3. Patient interview using standardised study instruments. | Low |
| | Outcome assessment by self-report or approach not described | High |
| 8. Completeness of outcome assessment | ≥90% of participants followed up for a sufficient duration of time to allow outcomes to occur | Low |
| | Complete follow-up for <90% of participants<br>or follow-up of insufficient duration<br>or completeness and duration of follow up not described | High |

**Supplementary Table 5.B.** ROB assessment tool results per included study.

| Author, year and country | Study design | Study period | Representati veness | Case identification | Sampling strategy | Specimen | Diagnostic test | Outcome assessment | Completeness of outcome assessment | ROB score (out of 9) |
|---|---|---|---|---|---|---|---|---|---|---|
| Ambrosioni, 2014, Switzerland | High | Low | High | High | High | Low | Low | Low | Low | 4 |
| Ansaldi, 2012, Italy | Low | High | High | Low | Low | Low | Low | Low | Low | 2 |
| Antalis, 2018, Greece | Low | High | High | Low | Low | Low | Low | Low | Low | 2 |
| Anton, 2016, Spain | Low | High | High | Low | Low | Low | Low | Low | Low | 2 |
| Arbefeville, 2017, USA | High | Low | High | High | High | Low | Low | Low | Low | 4 |
| Aronen, 2019, Finland | Low | Low | Low | Low | Low | Low | Low | Low | Low | 0 |
| Belongia, 2018, USA | Low | High | High | Low | Low | Low | Low | Low | Low | 2 |
| Borg, 2003, Germany | Low | Low | High | Low | Low | Low | Low | Low | Low | 1 |
| Camargo, 2008, USA | Low | High | High | Low | Low | Low | Low | Low | Low | 2 |
| Cameron, 2006, Australia | Low | Low | High | Low | Low | Low | Low | Low | Low | 1 |
| Campe, 2016, Germany | Low | High | High | Low | Low | Low | Low | Low | Low | 2 |
| Carrat, 2006, France | Low | High | High | Low | Low | Low | Low | Low | Low | 2 |
| Charles, 2008, Australia | Low | Low | High | Low | Low | Low | Low | Low | Low | 1 |
| Chasqueira, 2018, Portugal | Low | High | Low | Low | Low | Low | Low | Low | Low | 1 |
| Chatzis, 2018, Switzerland | High | Low | High | Low | Low | Low | Low | Low | Low | 2 |
| Clark, 2014, UK | Low | High | High | Low | Low | Low | Low | Low | Low | 2 |
| Damlaj, 2016, USA | High | Low | High | Low | High | Low | Low | Low | Low | 3 |
| D'Angelo, 2016, USA | High | Low | High | Low | High | Low | Low | Low | Low | 3 |
| De Serres, 2009 Canada | Low | High | High | Low | Low | Low | Low | Low | Low | 2 |
| Diaz-Decaro, 2016, USA | Low | High | Low | Low | Low | Low | Low | Low | Low | 1 |
| Dimopoulos, 2014, Greece | Low | Low | High | Low | Low | Low | Low | Low | Low | 1 |
| Falsey, 2005, USA | Low | High | Low | Low | Low | Low | High | Low | Low | 2 |
| Falsey, 2006 USA | Low | Low | Low | Low | Low | Low | Low | Low | Low | 0 |
| Falsey, 2014, Belgium, Canada, Czech Rep, Estonia, France, Germany, | Low | High | High | Low | High | Low | Low | Low | Low | 3 |

| **Author, year and country** | **Study design** | **Study period** | **Representati veness** | **Case identification** | **Sampling strategy** | **Specimen** | **Diagnostic test** | **Outcome assessment** | **Completeness of outcome assessment** | **ROB score (out of 9)** |
|---|---|---|---|---|---|---|---|---|---|---|
| Netherlands, Norway, Poland, Romania, UK | | | | | | | | | | |
| Garcia-Noblejas, 2015, Spain | High | Low | High | High | High | Low | Low | Low | High | 5 |
| Gaymard, 2018, France | High | High | High | High | High | Low | Low | Low | Low | 5 |
| Gaymard, 2019, France | High | High | High | High | High | Low | Low | Low | Low | 5 |
| Gilca, 2014, Canada | Low | High | High | Low | Low | Low | Low | Low | Low | 2 |
| Gimferrer, 2019, Spain | Low | High | High | Low | Low | Low | High | Low | Low | 3 |
| Gorcea, 2015, UK | High | Low | High | Low | High | Low | Low | Low | Low | 3 |
| Gorse, 2015, USA | Low | Low | Low | Low | Low | Low | Low | Low | Low | 0 |
| Graat, 2003, Netherlands | Low | Low | Low | Low | Low | Low | Low | Low | Low | 0 |
| Gueller, 2013, Germany | High | High | High | Low | Low | High | Low | Low | Low | 4 |
| Hequet, 2019, Switzerland | Low | High | Low | Low | Low | Low | Low | Low | Low | 1 |
| Hombrouck, 2012, Belgium | Low | High | High | Low | Low | Low | Low | Low | Low | 2 |
| Hopkins, 2008, Australia | Low | Low | Low | Low | Low | Low | Low | Low | Low | 0 |
| Huijts, 2018, Netherlands | Low | Low | High | Low | Low | High | Low | Low | Low | 2 |
| Hutchinson, 2007, Australia | Low | High | High | Low | Low | Low | Low | Low | Low | 2 |
| Iikura, 2015, Japan | Low | Low | High | Low | Low | Low | Low | Low | Low | 1 |
| Jahn, 2018, Switzerland | Low | Low | High | Low | Low | Low | Low | Low | Low | 1 |
| Jain, 2015, USA | Low | Low | High | Low | Low | Low | Low | Low | Low | 1 |
| Jeannoel, 2019, France | High | High | High | Low | High | Low | Low | Low | Low | 4 |
| Johnstone, 2014, Canada | Low | High | Low | Low | Low | Low | Low | High | Low | 2 |
| Juretschko, 2017, USA & Canada | Low | High | High | High | High | Low | Low | Low | Low | 4 |
| Katsurada, 2017, Japan | Low | Low | High | Low | Low | Low | Low | Low | Low | 1 |
| Khanna, 2008, Switzerland | High | Low | High | Low | High | Low | High | Low | Low | 4 |
| Kherad, 2010, Switzerland | Low | Low | High | Low | Low | Low | Low | Low | Low | 1 |
| Klein, 2007, Canada | Low | High | High | Low | Low | Low | High | Low | Low | 3 |
| Kumar, 2005, Canada | Low | Low | High | Low | Low | Low | Low | Low | Low | 1 |

| Author, year and country | Study design | Study period | Representati veness | Case identification | Sampling strategy | Specimen | Diagnostic test | Outcome assessment | Completeness of outcome assessment | ROB score (out of 9) |
|---|---|---|---|---|---|---|---|---|---|---|
| Lee, 2019, USA | High | Low | High | Low | High | Low | Low | Low | Low | 3 |
| Leibl, 2017, Switzerland | Low | High | High | Low | Low | Low | Low | Low | Low | 2 |
| Li, 2012, USA | High | Low | High | Low | Low | Low | Low | Low | Low | 2 |
| Lopez-Medrano, 2007, Spain | Low | High | Low | Low | Low | Low | High | Low | Low | 2 |
| Loubet, 2017, France | Low | High | High | Low | Low | Low | Low | Low | Low | 2 |
| Mahan, 2017, USA | High | Low | High | Low | High | Low | Low | Low | Low | 3 |
| Malosh, 2017, USA | Low | High | High | Low | Low | Low | Low | Low | Low | 2 |
| Mikulsa, 2014, Italy | Low | High | Low | Low | Low | Low | Low | Low | Low | 1 |
| Meerhoff, 2006, Netherlands, England, Scotland | Low | High | High | Low | Low | Low | Low | Low | Low | 2 |
| Milstone, 2006, USA | Low | High | Low | Low | Low | Low | Low | Low | Low | 1 |
| Minodier, 2014, France | Low | High | High | Low | Low | Low | Low | Low | Low | 2 |
| Ong, 2014, Netherlands | Low | High | High | Low | Low | Low | Low | Low | High | 3 |
| Paba, 2014, Italy | Low | Low | High | Low | Low | Low | Low | Low | Low | 1 |
| Pancer, 2011, Poland | Low | Low | High | High | High | Low | Low | Low | Low | 3 |
| Passi, 2019, Italy | Low | Low | High | Low | Low | Low | Low | Low | Low | 1 |
| Peghin, 2017, Spain | Low | Low | Low | Low | Low | Low | Low | Low | Low | 0 |
| Peyrani, 2012, USA | Low | High | High | Low | Low | Low | Low | Low | Low | 2 |
| Pilie, 2015, USA | High | Low | High | Low | High | Low | Low | Low | Low | 3 |
| Pinana, 2017, Spain | Low | Low | High | Low | Low | Low | Low | Low | Low | 1 |
| Price, 2019, Australia | High | Low | High | High | Low | Low | Low | Low | Low | 3 |
| Puig-Barbera, 2012, Spain | Low | High | High | Low | Low | Low | Low | Low | Low | 2 |
| Puzelli, 2009, Italy | Low | High | High | Low | Low | High | Low | Low | Low | 3 |
| Reid, 2017, Australia | High | High | High | Low | High | Low | Low | Low | Low | 4 |
| Renaud, 2013, USA | High | Low | High | Low | High | Low | Low | Low | Low | 3 |
| Roghmann, 2003, USA | Low | High | High | Low | Low | Low | Low | Low | Low | 2 |
| Rohde, 2003, Germany | Low | Low | High | Low | Low | Low | Low | Low | Low | 1 |

| Author, year and country | Study design | Study period | Representati veness | Case identification | Sampling strategy | Specimen | Diagnostic test | Outcome assessment | Completeness of outcome assessment | ROB score (out of 9) |
|---|---|---|---|---|---|---|---|---|---|---|
| Saez-Lopez, 2019, Portugal | Low | High | High | Low | Low | Low | Low | Low | Low | 2 |
| Sanghavi, 2012, USA | Low | High | High | High | High | High | Low | Low | Low | 5 |
| Saraya, 2017, Japan | Low | Low | Low | Low | Low | Low | Low | Low | Low | 0 |
| Schmidt, 2019, USA | High | Low | High | Low | High | Low | Low | Low | Low | 3 |
| Seemungal, 2001, UK | Low | Low | High | Low | Low | Low | High | Low | Low | 2 |
| Sellers, 2018, USA | Low | Low | High | Low | Low | Low | Low | Low | Low | 1 |
| Slade, 2017, USA | High | Low | Low | Low | High | Low | High | Low | Low | 3 |
| Snyder, 2017, USA | High | Low | High | Low | High | Low | Low | Low | Low | 3 |
| Souty, 2019, France | Low | High | High | Low | Low | Low | Low | Low | Low | 2 |
| Spahr, 2018, Switzerland | High | Low | High | High | High | Low | Low | Low | Low | 4 |
| Steensels, 2019, Belgium | High | Low | High | Low | High | Low | Low | Low | Low | 3 |
| Stolz, 2019, Switzerland | Low | Low | Low | Low | Low | Low | Low | Low | Low | 0 |
| Sundaram, 2014, USA | Low | High | High | Low | Low | Low | Low | Low | Low | 2 |
| Tanner, 2012, UK | High | High | High | High | High | Low | Low | Low | Low | 5 |
| Teh, 2015, Australia | High | Low | High | Low | High | Low | Low | Low | Low | 3 |
| Theodoropoulos, 2013, USA | High | Low | High | Low | High | High | Low | Low | Low | 4 |
| Thomas, 2019, USA | High | High | High | Low | High | Low | Low | Low | Low | 4 |
| Tramuto, 2016, Italy | High | Low | High | Low | Low | Low | Low | Low | Low | 2 |
| Van Beek, 2017, Netherlands | Low | High | Low | Low | Low | Low | Low | Low | Low | 1 |
| Varghese, 2018, Australia | Low | Low | High | Low | High | Low | Low | Low | Low | 2 |
| Visseaux, 2017, France | High | Low | High | Low | High | Low | Low | Low | Low | 3 |
| Walker, 2014, USA | High | Low | High | Low | High | Low | Low | Low | Low | 3 |
| Wansaula, 2016, USA | Low | Low | High | Low | Low | Low | Low | Low | High | 2 |
| Weinberg, 2010, USA | Low | Low | Low | Low | Low | Low | Low | Low | Low | 0 |
| Widmer, 2012, USA | Low | High | High | Low | Low | Low | Low | Low | Low | 2 |
| Widmer, 2014, USA | Low | Low | High | Low | Low | Low | Low | Low | Low | 1 |

| Author, year and country | Study design | Study period | Representativeness | Case identification | Sampling strategy | Specimen | Diagnostic test | Outcome assessment | Completeness of outcome assessment | ROB score (out of 9) |
|---|---|---|---|---|---|---|---|---|---|---|
| Yousaf, 2017, USA | High | Low | High | Low | High | Low | Low | Low | Low | 3 |
| Zambon, 2001, UK | Low | High | High | Low | High | Low | Low | Low | Low | 3 |

**Supplementary Table 6.** Characteristics of included studies.

| Num | Author, Year & country | Overall Sample Size | Age-group | Population according to study setting | Older adults or risk group | Study Years | Data collection | Study design | Outcome reported | Full citation |
|---|---|---|---|---|---|---|---|---|---|---|
| 1 | Ambrosioni, 2014, Switzerland | 1039 | >65 | Medically attended | Older adults | 2011-2012 | Continuous (annual) | Surveillance | RSV prevalence (Elderly) | Ambrosioni J, Bridevaux P-O, Wagner G, Mamin A, Kaiser L. Epidemiology of viral respiratory infections in a tertiary care centre in the era of molecular diagnosis, Geneva, Switzerland, 2011-2012. Clinical Microbiology and Infection 2014; 20(9): O578-O84. |
| 2 | Ansaldi, 2012, Italy | 2551 | ≥60 | Community cohort | Older adults | 2010-2011 | Seasonal | Cohort | Pneumonia proportion (Elderly); RSV proportion (Elderly) | Ansaldi F, De Florentiis D, Parodi V, et al. Bacterial carriage and respiratory tract infections in subjects ≥ 60 years during an influenza season: Implications for the epidemiology of Community Acquired Pneumonia and influenza vaccine effectiveness. Journal of Preventive Medicine and Hygiene 2012; 53(2): 94-7. |
| 3 | Antalis, 2018, Greece | 129 | >65 | Medically attended | Older adults | 2009-2015 | Seasonal | Surveillance | RSV proportion (Elderly) | Antalis E, Oikonomopoulou Z, Kottaridi C, et al. Mixed viral infections of the respiratory tract; an epidemiological study during consecutive winter seasons. Journal of Medical Virology 2018; 90(4): 663-70. |
| 4 | Anton, 2016, Spain | 339 | >65 | Medically attended, ILI | Older adults | 2006-2012 | Seasonal | Surveillance | RSV proportion (Elderly) | Anton A, Marcos MA, Torner N, et al. Virological surveillance of influenza and other respiratory viruses during six consecutive seasons from 2006 to 2012 in Catalonia, Spain. Clinical Microbiology and Infection 2016; 22(6): 564. |
| 5 | Arbefeville, 2017, USA | 614 | ≥60 | Medically attended | Older adults | 2014-2015 | Continuous (annual) | Surveillance | RSV proportion (Elderly) | Arbefeville S, Ferrieri P. Epidemiologic analysis of respiratory viral infections mainly in hospitalised children and adults in a Midwest University Medical Center after the implementation of a 14-virus multiplex nucleic acid amplification test. American Journal of Clinical Pathology 2017; 147(1): 43-9. |
| 6 | Aronen, 2019, Finland | 382 | ≥65 | Medically attended, inpatients | HR older adults, all with underlying comorbidities | 2007-2009 | Continuous (annual) | Cohort | Pneumonia proportion (Elderly); RSV proportion (Elderly) | Aronen M, Viikari L, Kohonen I, et al. Respiratory tract virus infections in the elderly with pneumonia. BMC geriatrics 2019; 19(1): 111. |

| Num | Author, Year & country | Overall Sample Size | Age-group | Population according to study setting | Older adults or risk group | Study Years | Data collection | Study design | Outcome reported | Full citation |
|---|---|---|---|---|---|---|---|---|---|---|
| 7 | Belongia, 2018, USA | 1832 | ≥60 | Medically attended | Older adults, cardiopulmonary, asthma, diabetes, immunodeficient (cause not specified), lung disease, cardiac disease | 2004-2016 | Seasonal | Cohort | Hospitalisation proportion (Elderly); ICU admission proportion (Elderly); RSV proportion (Elderly); RSV incidence (Elderly); Hospitalisation proportion (HR); RSV proportion (HR); RSV incidence (HR) | Belongia EA, King JP, Kieke BA, et al. Clinical features, severity, and incidence of RSV illness during 12 consecutive seasons in a community cohort of adults ≥60 years old. Open forum infectious diseases 2018; 5(12). |
| 8 | Borg, 2003, Germany | 125 | ≥18 | Medically attended, COPD inpatients | Cardiopulmonary | 1999-2001 | Continuous (annual) | Assay validation | RSV proportion (HR) | Borg I, Rohde G, Loseke S, et al. Evaluation of a quantitative real-time PCR for the detection of respiratory syncytial virus in pulmonary diseases. European Respiratory Journal 2003; 21(6): 944-51. |
| 9 | Camargo, 2008, USA | 76 | ≥50 | Medically attended, AECOPD | Cardiopulmonary, lung disease | 2003-2004 | Seasonal | Prospective case series | Hospitalisation proportion (HR); proportion (HR); RSV proportion (HR) | Camargo JCA, Ginde AA, Clark S, Cartwright CP, Falsey AR, Niewoehner DE. Viral pathogens in acute exacerbations of chronic obstructive pulmonary disease. Internal and Emergency Medicine 2008; 3(4): 355-9. |
| 10 | Cameron, 2006, Australia | 105 | >45 | Medically attended, AECOPD ICU ventilated patients | Cardiopulmonary, lung disease | 2000-2003 | Continuous (annual) | Prospective case series | RSV proportion (HR) | Cameron RJ, de Wit D, Welsh TN, Ferguson J, Grissell TV, Rye PJ. Virus infection in exacerbations of chronic obstructive pulmonary disease requiring ventilation. Intensive Care Med 2006; 32(7): 1022-9. |
| 11 | Campe, 2016, Germany | 28 | ≥60 | Medically attended, sentinel surveillance | Older adults | 2013-2013 | Seasonal | Surveillance | RSV proportion (Elderly) | Campe H, Heinzinger S, Hartberger C, Sing A. Clinical symptoms cannot predict influenza infection during the 2013 influenza season in Bavaria, Germany. Epidemiology and infection 2016; 144(5): 1045-51. |
| 12 | Carrat, 2006, France | 122 | ≥18 | Medically attended, critical care inpatients with cardiorespiratory failure | Cardiopulmonary | 2002-2014 | Seasonal | Prospective case series | RSV proportion (HR) | Carrat F, Leruez-Ville M, Tonnellier M, et al. A virologic survey of patients admitted to a critical care unit for acute cardiorespiratory failure. Intensive Care Medicine 2006; 32(1): 156-9. |

| Num | Author, Year & country | Overall Sample Size | Age-group | Population according to study setting | Older adults or risk group | Study Years | Data collection | Study design | Outcome reported | Full citation |
|---|---|---|---|---|---|---|---|---|---|---|
| 13 | Charles, 2008, Australia | 865 | ≥18; ≥65 | Medically attended (Emergency department), pneumonia patients | Older adults, cardiopulmonary, asthma, chronic kidney disease, diabetes, immunodeficient (defined as occurring in patients who took ≤10 mg prednisolone per day, who were pregnant, who had undergone splenectomy, or who had received an autologous stem cell transplant years earlier), dementia, care home | 2004-2006 | Continuous (annual) | Prospective case series | Case fatality rate (Elderly); Hospitalisation proportion (Elderly); RSV proportion (Elderly); RSV proportion (HR) | Charles PG, Whitby M, Fuller AJ, et al. The etiology of community-acquired pneumonia in Australia: why penicillin plus doxycycline or a macrolide is the most appropriate therapy. Clinical infectious diseases : an official publication of the Infectious Diseases Society of America 2008; 46(10): 1513-21. |
| 14 | Chasqueira, 2018, Portugal | 1022 | ≥59 | Care home | Care home | 2013-2014 | Seasonal | Cohort | RSV proportion (HR); RSV incidence (HR) | Chasqueira M-J, Paixao P, Rodrigues M-L, et al. Respiratory infections in elderly people: Viral role in a resident population of elderly care centers in Lisbon, winter 2013-2014. International journal of infectious diseases : IJID : official publication of the International Society for Infectious Diseases 2018; 69: 1-7. |
| 15 | Chatzis, 2018, Switzerland | 175 | ≥18 | Medically attended, RSV positive | Immunodeficient (allogeneic or autologous hematopoietic stem cell transplant recipients, solid organ transplant recipients, patients on cancer chemotherapy or long-term immunosuppression for any chronic disease) | 2005-2014 | Continuous (annual) | Retrospective case series | Case fatality rate (HR); Hospitalisation proportion (HR); ICU admission proportion (HR); LRTI proportion (HR); Pneumonia proportion (HR); URTI proportion (HR); Mechanical ventilation proportion (HR) | Chatzis O, Darbre S, Pasquier J, et al. Burden of severe RSV disease among immunocompromised children and adults: a 10 year retrospective study. BMC infectious diseases 2018; 18(1): 111. |
| 16 | Clark, 2014, UK | 780 | ≥18 | Medically attended, inpatients | Asthma, lung disease, cardiac disease | 2005-2008 | Seasonal | Assay validation | RSV proportion (HR) | Clark TW, Medina MJ, Batham S, Curran MD, Parmar S, Nicholson KG. Adults hospitalised with acute respiratory illness rarely have detectable bacteria in the absence of COPD or pneumonia; viral infection predominates in a large prospective UK sample. The Journal of infection 2014; 69(5): 507-15. |

| Num | Author, Year & country | Overall Sample Size | Age-group | Population according to study setting | Older adults or risk group | Study Years | Data collection | Study design | Outcome reported | Full citation |
|---|---|---|---|---|---|---|---|---|---|---|
| 17 | Damlaj, 2016, USA | 45 | ≥18 | Medically attended | Immunodeficient (allogeneic stem cell transplant recipients) | 2008-2014 | Continuous (annual) | Retrospective case series | Case fatality rate (HR); Hospitalisation proportion (HR); ICU admission proportion (HR); LRTI proportion (HR); URTI proportion (HR) | Damlaj M, Bartoo G, Cartin-Ceba R, et al. Corticosteroid use as adjunct therapy for respiratory syncytial virus infection in adult allogeneic stem cell transplant recipients. Transplant Infectious Disease 2016; 18(2): 216-26. |
| 18 | D'Angelo, 2016, USA | 118 | ≥50 | Medically attended, HSCT | Immunodeficient (allogeneic hematopoietic cell transplant recipients) | 2009-2013 | Continuous (annual) | Cohort | Case fatality rate (HR); Hospitalisation proportion (HR); LRTI proportion (HR); RSV proportion (HR) | D'Angelo CR, Kocherginsky M, Pisano J, et al. Incidence and predictors of respiratory viral infections by multiplex PCR in allogeneic hematopoietic cell transplant recipients 50 years and older including geriatric assessment. Leukemia and Lymphoma 2016; 57(8): 1807-13. |
| 19 | De Serres, 2009 Canada | 108 | ≥50 | Medically attended, AECOPD | Cardiopulmonary, lung disease | 2003-2004 | Seasonal | Prospective case series | RSV proportion (HR) | De Serres G, Lampron N, La Forge J, et al. Importance of viral and bacterial infections in chronic obstructive pulmonary disease exacerbations. Journal of Clinical Virology 2009; 46(2): 129-33. |
| 20 | Diaz-Decaro, 2016, USA | 52 | ≥18 | Care home | Care home | 2015-2015 | Seasonal (Summer) | Cohort | RSV proportion (HR) | Diaz-Decaro J, Launer B, Mckinnell JA, et al. Prevalence of respiratory viruses, including influenza, among nursing home residents and high-touch room surfaces. Open forum infectious diseases 2016; 3. |
| 21 | Dimopoulos, 2014, Greece | 247 | ≥18; ≥65 | Medically attended, AECOPD inpatients | Cardiopulmonary, lung disease | 2008-2010 | Continuous (annual) | Cohort | RSV proportion (HR) | Dimopoulos G, Tsiodras S, Lerikou M, et al. Viral profile of COPD exacerbations according to patients. Open Respiratory Medicine Journal 2014; 9(1): 1-8. |

| Num | Author, Year & country | Overall Sample Size | Age-group | Population according to study setting | Older adults or risk group | Study Years | Data collection | Study design | Outcome reported | Full citation |
|---|---|---|---|---|---|---|---|---|---|---|
| 22 | Falsey, 2005, USA | 2536 | ≥21, ≥65 | Community cohort & medically attended | Older adults, cardiopulmonary, lung disease, cardiac disease | 1999-2003 | Seasonal | Cohort | Case fatality rate (Elderly); Hospitalisation proportion (Elderly); RSV proportion (Elderly); RSV incidence (Elderly); Case fatality rate (HR); Emergency consultation proportion (HR); Hospitalisation proportion (HR); Outpatient proportion (HR); Pneumonia proportion (HR); RSV proportion (HR); RSV incidence (HR) | Falsey AR, Hennessey PA, Formica MA, Cox C, Walsh EE. Respiratory syncytial virus infection in elderly and HR adults. The New England journal of medicine 2005; 352(17): 1749-59. |
| 23 | Falsey, 2006 USA | 112 | ≥40 | Community cohort, COPD | Cardiopulmonary, lung disease | 2004-2005 | Continuous (annual) | Cohort | RSV proportion (HR) | Dimopoulos G, Tsiodras S, Lerikou M, et al. Viral profile of COPD exacerbations according to patients. Open Respiratory Medicine Journal 2014; 9(1): 1-8. |
| 24 | Falsey, 2014, Belgium, Canada, Czech Rep, Estonia, France, Germany, Netherlands, Norway, Poland, Romania, UK | 404 | ≥65 | Community cohort, ILI | Older adults | 2008-2010 | Seasonal | Trial | RSV proportion (Elderly); RSV proportion (Elderly) | Falsey AR, McElhaney JE, Beran J, et al. Respiratory syncytial virus and other respiratory viral infections in older adults with moderate to severe influenza-like illness. The Journal of infectious diseases 2014; 209(12): 1873-81. |
| 25 | Garcia-Noblejas, 2015, Spain | 211 | ≥18 | Medically attended, haematology patients | Immunodeficient (haematological disease patients) | 2012-2014 | Continuous (annual) | Retrospective case series | Case fatality rate (HR); LRTI proportion (HR); RSV proportion (HR); URTI proportion (HR) | Garcia-Noblejas A, Lorenzo A, Cardenoso L, Villanueva M, De La Camara R. Community acquiered respiratory virus in adults patients with hematological disease: Clinical characteristics and outcome in RSV and HPIV infection. Haematologica 2015; 100: 296-7. |

| Num | Author, Year & country | Overall Sample Size | Age-group | Population according to study setting | Older adults or risk group | Study Years | Data collection | Study design | Outcome reported | Full citation |
|---|---|---|---|---|---|---|---|---|---|---|
| 26 | Gaymard, 2018, France | 4232 | >65 | Medically attended | Older adults | 2010-2014 | Seasonal | Surveillance | RSV proportion (Elderly) | Gaymard A, Bouscambert-Duchamp M, Pichon M, et al. Genetic characterisation of respiratory syncytial virus highlights a new BA genotype and emergence of the ON1 genotype in Lyon, France, between 2010 and 2014. Journal of clinical virology : the official publication of the Pan American Society for Clinical Virology 2018; 102: 12-8. |
| 27 | Gaymard, 2019, France | 6931 | >65 | Medically attended | Older adults | 2014-2018 | Seasonal | Surveillance | RSV proportion (Elderly) | Gaymard A, Pichon M, Ibranosyan M, et al. Epidemiology of respiratory syncytial virus circulating in Lyon, France, between 2014 and 2018. Virologie 2019; 23(2): 93. |
| 28 | Gilca, 2014, Canada | 474 | ≥65 | Medically attended, inpatients | Older adults | 2012-2013 | Seasonal | Trial | RSV proportion (Elderly) | Gilca R, Amini R, Douville-Fradet M, et al. Other respiratory viruses are important contributors to adult respiratory hospitalisations and mortality even during peak weeks of the influenza season. Open forum infectious diseases 2014; 1(2). |
| 29 | Gimferrer, 2019, Spain | 6534 | >64 | Medically attended | Older adults | 2013-2018 | Continuous (annual) | Surveillance | RSV proportion (Elderly) | Gimferrer L, Vila J, Pinana M, et al. Virological surveillance of human respiratory syncytial virus A and B at a tertiary hospital in Catalonia (Spain) during five consecutive seasons (2013-2018). Future Microbiology 2019; 14(5): 373-81. |
| 30 | Gorcea, 2015, UK | 407 | ≥18 | Medically attended, HSCT | Immunodeficient (haematopoietic stem cell transplant recipient) | 2010-2014 | Continuous (annual) | Retrospective case series | Hospitalisation proportion (HR); ICU admission proportion (HR) | Gorcea CM, Tholouli E, Turner A, Flaum N, Dignan F. The clinical and financial impact of respiratory syncytial virus infection post-haematopoietic stem cell transplantation. Bone Marrow Transplantation 2015; 50: S194-S5. |
| 31 | Gorse, 2015, USA | 100 | ≥60 | Community cohort, patients with cardiopulmo nary disease | Cardiopulmonary | 2009-2013 | Continuous (annual) | Cohort | RSV proportion (HR) | Gorse GJ, Donovan MM, Patel GB, Balasubramanian S, Lusk RH. Coronavirus and Other Respiratory Illnesses Comparing Older with Young Adults. American Journal of Medicine 2015; 128(11): 1251e11-e20. |
| 32 | Graat, 2003, Netherlands | 97 | ≥60 | Community cohort | Older adults | 1998-2000 | Continuous (annual) | Case control | RSV proportion (Elderly) | Graat JM, Schouten EG, Heijnen ML, et al. A prospective, community-based study on virologic assessment among elderly people with and without symptoms of acute respiratory infection. J Clin Epidemiol 2003; 56(12): 1218-23. |

| Num | Author, Year & country | Overall Sample Size | Age-group | Population according to study setting | Older adults or risk group | Study Years | Data collection | Study design | Outcome reported | Full citation |
|---|---|---|---|---|---|---|---|---|---|---|
| 33 | Gueller, 2013, Germany | 29 | ≥18 | Medically attended, HSCT | Immunodeficient (haematopoietic stem cell transplant recipient) | 2008-2009 | Seasonal | Retrospective case series | Bronchitis proportion (HR); LRTI proportion (HR); Oxygen therapy proportion (HR); Pneumonia proportion (HR); RSV proportion (HR); URTI proportion (HR) | Gueller S, Duenzinger U, Wolf T, et al. Successful systemic high-dose ribavirin treatment of respiratory syncytial virus-induced infections occurring pre-engraftment in allogeneic hematopoietic stem cell transplant recipients. Transplant Infectious Disease 2013; 15(4): 435-40. |
| 34 | Hequet, 2019, Switzerland | 509 | ≥18 | Care home | Care home | 2016-2018 | Seasonal | Cohort | Antibiotic use proportion (HR); Case fatality rate (HR); Hospitalisation proportion (HR); Oxygen therapy proportion (HR); RSV proportion (HR) | Hequet D, Rochat A, Petignat C. Respiratory syncytial virus, a threat for nursing homes residents? Antimicrobial Resistance and Infection Control 2019; 8. |
| 35 | Hombrouck, 2012, Belgium | 18 | ≥65 | Medically attended, influenza negative ILI cases | Older adults | 2009-2010 | Seasonal | Surveillance | RSV proportion (Elderly) | Hombrouck A, Sabbe M, Van Casteren V, et al. Viral aetiology of influenza-like illness in Belgium during the influenza A(H1N1)2009 pandemic. European journal of clinical microbiology & infectious diseases : official publication of the European Society of Clinical Microbiology 2012; 31(6): 999-1007. |
| 36 | Hopkins, 2008, Australia | 89 | ≥18 | Community cohort, SOT | Immunodeficient (lung transplant recipients) | 2003-2006 | Continuous (annual) | Cohort | Case fatality rate (HR); Respiratory failure proportion (HR); RSV proportion (HR); URTI proportion (HR) | Hopkins P, McNeil K, Kermeen F, et al. Human metapneumovirus in lung transplant recipients and comparison to respiratory syncytial virus. American journal of respiratory and critical care medicine 2008; 178(8): 876-81. |
| 37 | Huijts, 2018, Netherlands | 84496 | ≥65 | Community cohort, pneumonia patients | Older adults | 2008-2013 | Continuous (annual) | Trial | RSV proportion (Elderly); RSV incidence (Elderly) | Huijts SM, Coenjaerts FEJ, Bolkenbaas M, et al. The impact of 13-valent pneumococcal conjugate vaccination on virus-associated community-acquired pneumonia in elderly: Exploratory analysis of the CAPiTA trial. Clinical microbiology and infection : the official publication of the European Society of Clinical Microbiology and Infectious Diseases 2018; 24(7): 764-70. |

| Num | Author, Year & country | Overall Sample Size | Age-group | Population according to study setting | Older adults or risk group | Study Years | Data collection | Study design | Outcome reported | Full citation |
|---|---|---|---|---|---|---|---|---|---|---|
| 38 | Hutchinson, 2007, Australia | 92 | ≥18 | Community cohort, AECOPD patients | Cardiopulmonary, lung disease | 2003-2005 | Seasonal | Case control | RSV proportion (HR); RSV incidence (HR) | Hutchinson AF, Ghimire AK, Thompson MA, et al. A community-based, time-matched, case-control study of respiratory viruses and exacerbations of COPD. Respiratory Medicine 2007; 101(12): 2472-81. |
| 39 | Iikura, 2015, Japan | 48 | ≥18 | Medically attended, asthma exacerbation inpatients | Asthma | 2011-2012 | Continuous (annual) | Prospective case series | RSV proportion (HR) | Iikura M, Hojo M, Koketsu R, et al. The importance of bacterial and viral infections associated with adult asthma exacerbations in clinical practice. PloS one 2015; 10(4): e0123584. |
| 40 | Jahn, 2018, Switzerland | 1303 | ≥18 | Medically attended, immunocompromised | Immunodeficient (haematological disease patients and solid organ transplant recipients) | 2009-2017 | Continuous (annual) | Prospective case series | RSV proportion (HR) | Jahn K, Schumann D, Tamm M, et al. Respiratory viral infection in immunocompromised patients. Respiration 2018; 95(6): 506-7. |
| 41 | Jain, 2015, USA | 805 | ≥65 | Medically attended, pneumonia inpatients | Older adults | 2010-2012 | Seasonal | Cohort | RSV proportion (Elderly) | Jain S, Self WH, Wunderink RG, et al. Community-Acquired Pneumonia Requiring Hospitalization among U.S. Adults. The New England journal of medicine 2015; 373(5): 415-27. |
| 42 | Jeannoel, 2019, France | 14792 | ≥18, ≥65 | Medically attended | Older adults, immunodeficient (reason not specified) | 2013-2016 | Seasonal | Retrospective case series | Case fatality rate (Elderly); ICU admission proportion (Elderly); Pneumonia proportion (Elderly); Antibiotic use proportion (HR); ARDS proportion (HR); ARDS proportion (HR); ARDS proportion (HR); Case fatality rate (HR); ICU admission proportion (HR) | Jeannoel M, Lina G, Rasigade JP, Lina B, Morfin F, Casalegno JS. Microorganisms associated with respiratory syncytial virus pneumonia in the adult population. European Journal of Clinical Microbiology and Infectious Diseases 2019; 38(1): 157-60. |
| 43 | Johnstone, 2014, Canada | 1072 | ≥65 | Care home | Care home | 2009-2012 | Seasonal | Cohort | RSV proportion (HR) | Johnstone J, Parsons R, Botelho F, et al. Immune biomarkers predictive of respiratory viral infection in elderly nursing home residents. PLoS ONE 2014; 9(10): e108481. |

| Num | Author, Year & country | Overall Sample Size | Age-group | Population according to study setting | Older adults or risk group | Study Years | Data collection | Study design | Outcome reported | Full citation |
|---|---|---|---|---|---|---|---|---|---|---|
| 44 | Juretschko, 2017, USA & Canada | 732 | >65 | Medically attended | Older adults | 2015-2016 | Seasonal | Assay validation | RSV proportion (Elderly) | Juretschko S, Mahony J, Buller RS, et al. Multicenter clinical evaluation of the luminex aries flu A/B and RSV assay for pediatric and adult respiratory tract specimens. Journal of clinical microbiology 2017; 55(8): 2431-8. |
| 45 | Katsurada, 2017, Japan | 2037 | ≥65 | Medically attended, pneumonia | Older adults | 2011-2014 | Continuous (annual) | Surveillance | RSV proportion (Elderly) | Katsurada N, Suzuki M, Aoshima M, et al. The impact of virus infections on pneumonia mortality is complex in adults: a prospective multicentre observational study. BMC infectious diseases 2017; 17(1): 755. |
| 46 | Khanna, 2008, Switzerland | 34 | ≥18 | Medically attended, haematology patients | Immunodeficient (haematological disease patients) | 2002-2007 | Continuous (annual) | Retrospective case series | Case fatality rate (HR); Hospitalisation proportion (HR); ICU admission proportion (HR); LRTI proportion (HR); Respiratory failure proportion (HR); URTI proportion (HR); Mechanical ventilation proportion (HR) | Khanna N, Widmer AF, Decker M, et al. Respiratory syncytial virus infection in patients with hematological diseases: Single-center study and review of the literature. Clinical Infectious Diseases 2008; 46(3): 402-12. |
| 47 | Kherad, 2010, Switzerland | 86 | ≥60 | Medically attended, AECOPD inpatients | Cardiopulmonary, lung disease | 2007-2008 | Continuous (annual) | Cohort | RSV proportion (HR) | Kherad O, Kaiser L, Bridevaux P-O, et al. Upper-respiratory viral infection, biomarkers, and COPD exacerbations. Chest 2010; 138(4): 896-904. |
| 48 | Klein, 2007, Canada | 50 | ≥18 | Medically attended, HIV | Immunodeficient (HIV patients) | 2003-2006 | Seasonal | Surveillance | RSV proportion (HR) | Klein MB, Lu Y, DelBalso L, Cote S, Boivin G. Influenza virus infection is a primary cause of febrile respiratory illness in HIV-infected adults, despite vaccination. Clinical Infectious Diseases 2007; 45(2): 234-40. |
| 49 | Kumar, 2005, Canada | 50 | ≥18 | Medically attended, SOT patients | Immunodeficient (lung transplant recipients) | 2001-2003 | Continuous (annual) | Cohort | LRTI proportion (HR); Pneumonia proportion (HR); RSV proportion (HR); URTI proportion (HR) | Kumar D, Erdman D, Keshavjee S, et al. Clinical impact of community-acquired respiratory viruses on bronchiolitis obliterans after lung transplant. American Journal of Transplantation 2005; 5(8): 2031-6. |

| Num | Author, Year & country | Overall Sample Size | Age-group | Population according to study setting | Older adults or risk group | Study Years | Data collection | Study design | Outcome reported | Full citation |
|---|---|---|---|---|---|---|---|---|---|---|
| 50 | Lee, 2019, USA | 326 | ≥18; >65 | Medically attended, RSV-positive inpatients | Older adults, cardiopulmonary, asthma, immunodeficient (reason not specified) | 2014-2016 | Continuous (annual) | Retrospective case series | Antibiotic use proportion (HR) | Lee N, Walsh EE, Sander I, et al. Delayed Diagnosis of Respiratory Syncytial Virus Infections in Hospitalized Adults: Individual Patient Data, Record Review Analysis and Physician Survey in the United States. The Journal of infectious diseases 2019; 220(6): 969-79. |
| 51 | Leibl, 2017, Switzerland | 100 | ≥18 | Medically attended, SOT patients | Immunodeficient (lung transplant recipients) | 2016-2017 | Seasonal | Assay validation | RSV proportion (HR) | Leibl M, Robinson C, Boeni J, et al. Diagnostic performance of rapid and standard polymerase chain reaction laboratory tests for influenza and respiratory syncytial virus detection in nasopharyngeal swabs from symptomatic lung transplant recipients. Chest 2017; 152(4): A1110. |
| 52 | Li, 2012, USA | 21 | ≥18 | Medically attended, SOT patients | Immunodeficient (lung transplant recipients | 2006-2010 | Continuous (annual) | Retrospective case series | Case fatality rate (HR); LRTI proportion (HR); Oxygen therapy proportion (HR); URTI proportion (HR) | Li L, Avery R, Budev M, Mossad S, Danziger-Isakov L. Oral versus inhaled ribavirin therapy for respiratory syncytial virus infection after lung transplantation. Journal of Heart and Lung Transplantation 2012; 31(8): 839-44. |
| 53 | Lopez-Medrano, 2007, Spain | 152 | ≥18 | Community cohort, SOT patients | Immunodeficient (solid organ transplant patients) | 2002-2003 | Seasonal | Cohort | Case fatality rate (HR); RSV proportion (HR); RSV incidence (HR) | Lopez-Medrano F, Aguado JM, Lizasoain M, et al. Clinical implications of respiratory virus infections in solid organ transplant recipients: A prospective study. Transplantation 2007; 84(7): 851-6. |
| 54 | Loubet, 2017, France | 1452 | ≥65 | Medically attended, ILI | Older adults, cardiopulmonary, asthma, CKD, diabetes, immunodeficient (immunosuppressive treatment) | 2012-2015 | Seasonal | Prospective case series | Case fatality rate (Elderly); RSV proportion (Elderly); Case fatality rate (HR) | Loubet P, Lenzi N, Valette M, et al. Clinical characteristics and outcome of respiratory syncytial virus infection among adults hospitalized with influenza-like illness in France. Clinical Microbiology and Infection 2017; 23(4): 253-9. |
| 55 | Mahan, 2017, USA | 66 | ≥18 | Medically attended, SOT patients | Immunodeficient (lung transplant recipients | 2013-2014 | Continuous (annual) | Retrospective case series | RSV proportion (HR); RSV incidence (HR) | Mahan L, Mohanka M, Mullins J, et al. Community-acquired respiratory virus infections during the first year after lung transplantation. Chest 2017; 152(4): A1108. |
| 56 | Malosh, 2017, USA | 1261 | ≥18; >65 | Medically attended | Older adults, lung and cardiovascular disease | 2014-2016 | Seasonal | Prospective case series | Case fatality rate (Elderly); RSV proportion (Elderly) | Malosh RE, Martin ET, Callear AP, et al. Respiratory syncytial virus hospitalization in middle-aged and older adults. Journal of Clinical Virology 2017; 96: 37-43. |
| 57 | Mikulska, 2014, USA | 193 | >=18 | Medically attended | Immunodeficient XX | 2011 | Seasonal | Cohort | Case fatality rate (HR); RSV prevalence (HR) | Mikulska M, Del Bono V, Gandolfo N, et al. Epidemiology of viral respiratory tract infections in an outpatient haematology facility. Ann Hematol 2014; 93(4): 669-76. |

| Num | Author, Year & country | Overall Sample Size | Age-group | Population according to study setting | Older adults or risk group | Study Years | Data collection | Study design | Outcome reported | Full citation |
|---|---|---|---|---|---|---|---|---|---|---|
| 58 | Meerhoff, 2006, Netherlands, England, Scotland | 110 | ≥65 | Medically attended, ILI | Older adults | 2002-2003 | Seasonal | Surveillance | Case fatality rate (HR); RSV proportion (HR) | Meerhoff TJ, Fleming D, Smith A, Mosnier A, van Gageldonk-Lafeber AB, W.J. P. Surveillance recommendations based on an exploratory analysis of respiratory syncytial virus reports derived from the European Influenza Surveillance System. BMC infectious diseases 2006; 6: 128. |
| 59 | Milstone, 2006, USA | 50 | ≥18 | Community cohort, SOT patients | Immunodeficient (lung transplant recipients | 1999-2000 | Seasonal | Cohort | Case fatality rate (HR); Hospitalisation proportion (HR); proportion (HR); LRTI proportion (HR); Pneumonia proportion (HR); RSV proportion (HR); RSV incidence (HR) | Milstone AP, Brumble LM, Barnes J, et al. A single-season prospective study of respiratory viral infections in lung transplant recipients. European Respiratory Journal 2006; 28(1): 131-7. |
| 60 | Minodier, 2014, France | 10 | ≥65 | Medically attended, ILI | Older adults | 2012-2013 | Seasonal | Surveillance | RSV proportion (Elderly) | Minodier L, Arena C, Heuze G, et al. Epidemiology and viral etiology of the influenza-like illness in Corsica during the 2012-2013 Winter: an analysis of several sentinel surveillance systems. PLoS One 2014; 9(6): e100388. |
| 61 | Ong, 2014, Netherlands | 158 | ≥18 | Medically attended, ICU patients with respiratory failure | Cardiopulmonary, immunodeficient (immunosuppressive treatment) | 2010-2013 | Seasonal | Prospective case series | Case fatality rate (HR); RSV proportion (HR) | Ong DSY, Faber TE, Klein Klouwenberg PMC, et al. Respiratory syncytial virus in critically ill adult patients with community-acquired respiratory failure: A prospective observational study. Clinical Microbiology and Infection 2014; 20(8): O505-O7. |
| 62 | Paba, 2014, Italy | 107 | ≥60 | Medically attended, ILI | Older adults, immunodeficient (reason not specified) | 2009-2011 | Continuous (annual) | Assay validation | RSV proportion (Elderly); RSV proportion (HR) | Paba P, Farchi F, Mortati E, et al. Screening of respiratory pathogens by Respiratory Multi Well System (MWS) r-geneTM assay in hospitalized patients. New Microbiologica 2014; 37(2): 231-6. |
| 63 | Pancer, 2011, Poland | 96 | >18 | Medically attended, chronic respiratory diseases | Cardiopulmonary | 2008-2010 | Continuous (annual) | Prospective case series | RSV proportion (HR) | Pancer K, Ciacka A, Gut W, et al. Infections caused by RSV among children and adults during two epidemic seasons. Polish Journal of Microbiology 2011; 60(3): 253-8. |

| Num | Author, Year & country | Overall Sample Size | Age-group | Population according to study setting | Older adults or risk group | Study Years | Data collection | Study design | Outcome reported | Full citation |
|---|---|---|---|---|---|---|---|---|---|---|
| 64 | Passi, 2019, Italy | 151 | ≥18 | Medically attended, haematology patients | Immunodeficient (haematological patients with acute leukemia, lymphoma, myeloma or other haem diseases) | 2011-2019 | Continuous (annual) | Surveillance | RSV proportion (HR) | Passi A, Pagani C, Gramegna D, et al. Respiratory viruses infections are a significant clinical problem in haematological patients with underestimated adverse outcome: A single institution 9-years experience. Haematologica 2019; 104: 29-30. |
| 65 | Peghin, 2017, Spain | 98 | ≥18 | Community cohort, SOT patients | Immunodeficient (lung transplant recipients | 2009-2014 | Continuous (annual) | Cohort | Bronchitis proportion (HR); Case fatality rate (HR); Hospitalisation proportion (HR); ICU admission proportion (HR); LRTI proportion (HR); Pneumonia proportion (HR); RSV proportion (HR); RSV incidence (HR); URTI proportion (HR) | Peghin M, Hirsch HH, Len O, et al. Epidemiology and Immediate Indirect Effects of Respiratory Viruses in Lung Transplant Recipients: A 5-Year Prospective Study. American Journal of Transplantation 2017; 17(5): 1304-12. |
| 66 | Peyrani, 2012, USA | 48 | ≥60 | Medically attended, AECOPD & pneumonia inpatients | Cardiopulmonary | 2010-2011 | Seasonal | Trial | RSV proportion (HR) | Peyrani P, Nahas A, Giovini V, et al. Respiratory viruses are significant etiologic agents in hospitalized patients with lower respiratory tract infections: Results from the rapid empiric treatment with oseltamivir study (RETOS). American Journal of Respiratory and Critical Care Medicine 2012; 185. |
| 67 | Pilie, 2015, USA | 69 | ≥18 | Medically attended, RSV-positive HSCT & SOT inpatients | Immunodeficient (hematopoietic stem cell transplant and solid organ transplant recipients) | 2009-2012 | Continuous (annual) | Retrospective case series | Case fatality rate (HR); ICU admission proportion (HR); LRTI proportion (HR); Pneumonia proportion (HR); URTI proportion (HR); Mechanical ventilation proportion (HR) | Pilie P, Werbel WA, Riddell J, Shu X, Schaubel D, Gregg KS. Adult patients with respiratory syncytial virus infection: Impact of solid organ and hematopoietic stem cell transplantation on outcomes. Transplant Infectious Disease 2015; 17(4): 551-7. |

| Num | Author, Year & country | Overall Sample Size | Age-group | Population according to study setting | Older adults or risk group | Study Years | Data collection | Study design | Outcome reported | Full citation |
|---|---|---|---|---|---|---|---|---|---|---|
| 68 | Pinana, 2017, Spain | 35 | ≥18 | Medically attended, RSV-positive HSCT patients | Immunodeficient (allogeneic hematopoietic stem cell transplantation) recipients) | 2013-2015 | Continuous (annual) | Trial | Antibiotic use proportion (HR); Hospitalisation proportion (HR); LRTI proportion (HR); URTI proportion (HR) | Pinana JL, Hernandez-Boluda JC, Calabuig M, et al. A risk-adapted approach to treating respiratory syncytial virus and human parainfluenza virus in allogeneic stem cell transplantation recipients with oral ribavirin therapy: A pilot study. Transplant infectious disease: an official journal of the Transplantation Society 2017; 19(4). |
| 69 | Price, 2019, Australia | 7777 | ≥65 | Medically attended | Older adults | 2002-2015 | Continuous with gaps (annual) | Surveillance | RSV proportion (Elderly) | Price OH, Sullivan SG, Sutterby C, Druce J, Carville KSA-POH, http://orcid.org/---735X O. Using routine testing data to understand circulation patterns of influenza A, respiratory syncytial virus and other respiratory viruses in Victoria, Australia. Epidemiology and infection 2019; 147: e221. |
| 70 | Puig-Barbera, 2012, Spain | 799 | ≥60 | Medically attended, ILI inpatients | Older adults, cardiopulmonary, CKD, diabetes, immunodeficient (immunosuppressive treatments) | 2010-2011 | Seasonal | Vaccine efficacy study | Case fatality rate (Elderly); ICU admission proportion (Elderly); Pneumonia proportion (Elderly); RSV proportion (Elderly); RSV proportion (HR) | Puig-Barbera J, Diez-Domingo J, Arnedo-Pen aA, et al. Effectiveness of the 2010-2011 seasonal influenza vaccine in preventing confirmed influenza hospitalizations in adults: A case-case comparison, case-control study. Vaccine 2012; 30(39): 5714-20. |
| 71 | Puzelli, 2009, Italy | 37 | ≥65 | Medically attended, ILI | Older adults | 2004-2007 | Seasonal | Surveillance | RSV proportion (Elderly) | Puzelli S, Valdarchi C, Ciotti M, et al. Viral causes of influenza-like illness: Insight from a study during the winters 2004-2007. Journal of Medical Virology 2009; 81(12): 2066-71. |
| 72 | Reid, 2017, Australia | 184 | ≥18 | Medically attended, AECOPD | Cardiopulmonary, lung disease | 2015-2015 | Seasonal | Retrospective case series | RSV proportion (HR) | Reid DW, Tse T, Jong T, Masel P, Smith DJ. Profile of bacterial and viral pathogens in patients presenting to hospital with an acute exacerbation of copd. Respirology 2017; 22: 138. |
| 73 | Renaud, 2013, USA | 23 | ≥18 | Medically attended, RSV positive | Immunodeficient (haematopoietic stem cell transplant recipients) | 2007-2011 | Continuous (annual) | Retrospective case series | Case fatality rate (HR); Respiratory failure proportion (HR) | Renaud C, Xie H, Seo S, et al. Mortality rates of human Metapneumovirus and respiratory syncytial virus lower respiratory tract infections in hematopoietic cell transplantation recipients. Biology of Blood and Marrow Transplantation 2013; 19(8): 1220-6. |

| Num | Author, Year & country | Overall Sample Size | Age-group | Population according to study setting | Older adults or risk group | Study Years | Data collection | Study design | Outcome reported | Full citation |
|---|---|---|---|---|---|---|---|---|---|---|
| 74 | Roghmann, 2003, USA | 62 | ≥18 | Community cohort, HSCT & bone marrow patients | Immunodeficient (bone marrow or peripheral blood stem cell transplant recipients) | 2001-2001 | Seasonal | Cohort | Hospitalisation proportion (HR); LRTI proportion (HR); Respiratory failure proportion (HR); RSV proportion (HR); RSV incidence (HR); URTI proportion (HR) | Roghmann M, Ball K, Erdman D, Lovchik J, Anderson LJ, Edelman R. Active surveillance for respiratory virus infections in adults who have undergone bone marrow and peripheral blood stem cell transplantation. Bone Marrow Transplantation 2003; 32(11): 1085-8. |
| 75 | Rohde, 2003, Germany | 85 | ≥18 | Medically attended, AECOPD inpatients | Cardiopulmonary, lung disease | 1998-1999 | Continuous (annual) | Cohort | RSV proportion (HR) | Rohde G, Wiethege A, Borg I, et al. Respiratory viruses in exacerbations of chronic obstructive pulmonary disease requiring hospitalisation: A case-control study. Thorax 2003; 58(1): 37-42. |
| 76 | Saez-Lopez, 2019, Portugal | 952 | ≥65 | Medically attended, ILI | Older adults | 2010-2018 | Seasonal | Surveillance | RSV proportion (Elderly) | Saez-Lopez E, Pechirra P, Costa I, et al. Performance of surveillance case definitions for respiratory syncytial virus infections through the sentinel influenza surveillance system, Portugal, 2010 to 2018. Euro surveillance : bulletin Europeen sur les maladies transmissibles = European communicable disease bulletin 2019 ; 24(45). |
| 77 | Sanghavi, 2012, USA | 105 | ≥18 | Medically attended, SOT | Immunodeficient (solid organ transplant recipients) | 2006-2007 | Seasonal | Prospective case series | LRTI proportion (HR); RSV proportion (HR); URTI proportion (HR) | Sanghavi SK, Bullotta A, Husain S, Rinaldo CR. Clinical evaluation of multiplex real-time PCR panels for rapid detection of respiratory viral infections. Journal of Medical Virology 2012; 84(1): 162-9. |
| 78 | Saraya, 2017, Japan | 106 | ≥18 | Medically attended, asthma patients | Asthma | 2012-2015 | Continuous (annual) | Cross-sectional | Hospitalisation proportion (HR); RSV proportion (HR) | Saraya T, Kimura H, Kurai D, Ishii H, Takizawa H. The molecular epidemiology of respiratory viruses associated with asthma attacks. Medicine (United States) 2017; 96(42): e8204. |
| 79 | Schmidt, 2019, USA | 489 | ≥18; >65 | Medically attended, RSV-positive inpatients | Older adults, CKD, diabetes, immunodeficient (immunosuppressive treatment), lung disease, cardiac disease | 2009-201 | Continuous (annual) | Cohort | Case fatality rate (HR); Discharge to care proportion (HR); ICU admission proportion (HR); Mechanical ventilation proportion (HR) | Schmidt H, Das A, Nam H, Yang A, Ison MGA-I, Michael G., http://orcid.org/--- O. Epidemiology and outcomes of hospitalized adults with respiratory syncytial virus: A 6-year retrospective study. Influenza and other respiratory viruses 2019; 13(4): 331-8. |

| Num | Author, Year & country | Overall Sample Size | Age-group | Population according to study setting | Older adults or risk group | Study Years | Data collection | Study design | Outcome reported | Full citation |
|---|---|---|---|---|---|---|---|---|---|---|
| 80 | Seemungal, 2001, UK | 83 | ≥18 | Community cohort, AECOPD | Cardiopulmonary, lung disease | | Continuous (annual) | Cohort | RSV proportion (HR) | Seemungal T, Harper-Owen R, Bhowmik A, et al. Respiratory viruses, symptoms, and inflammatory markers in acute exacerbations and stable chronic obstructive pulmonary disease. American Journal of Respiratory and Critical Care Medicine 2001; 164(9): 1618-23. |
| 81 | Sellers, 2018, USA | 70 | ≥18 | Medically attended, HIV | Immunodeficient (HIV patients) | 2015-2018 | Continuous (annual) | Cross-sectional | RSV proportion (HR) | Sellers S, Dover K, Wohl DA, Miller M, Dittmer D, Fischer W. The burden of respiratory viral illness in HIV-infected patients. Open forum infectious diseases 2018; 5: S670. |
| 82 | Slade, 2017, USA | 104 | ≥18 | Community cohort, HSCT patients | Immunodeficient (haploidentical peripheral blood hematopoietic cell transplant recipients) | 2009-2015 | Continuous (annual) | Cohort | LRTI proportion (HR); RSV proportion (HR); RSV incidence (HR); URTI proportion (HR) | Slade M, Goldsmith S, Romee R, et al. Epidemiology of infections following haploidentical peripheral blood hematopoietic cell transplantation. Transplant Infectious Disease 2017; 19(1): e12629. |
| 83 | Snyder, 2017, USA | 250 | ≥18 | Medically attended, AECOPD | Cardiopulmonary, lung disease | 2013-2014 | Continuous (annual) | Retrospective case series | RSV proportion (HR) | Snyder ME, Aaron CP, Regalbuto R, et al. Impact of virulent viral pathogens on hospital length of stay and readmissions after an acute exacerbation of chronic obstructive pulmonary disease. American Journal of Respiratory and Critical Care Medicine 2017; 195. |
| 84 | Souty, 2019, France | 337 | ≥65 | Medically attended, ILI | Older adults | 2015-2017 | Seasonal | Surveillance | RSV proportion (Elderly) | Souty C, Masse S, Valette M, et al. Baseline characteristics and clinical symptoms related to respiratory viruses identified among patients presenting with influenza-like illness in primary care. Clinical Microbiology and Infection 2019; 25(9): 1147-53. |
| 85 | Spahr, 2018, Switzerland | 33 | ≥18 | Community cohort, HSCT patients | Immunodeficient (allogeneic hematopoietic cell transplant recipients) | 2010-2014 | Continuous (annual) | Retrospective case series | Case fatality rate (HR); Hospitalisation proportion (HR); ICU admission proportion (HR); LRTI proportion (HR); URTI proportion (HR); Mechanical ventilation proportion (HR) | Spahr Y, Tschudin-Sutter S, Baettig V, et al. Community-acquired respiratory paramyxovirus infection after allogeneic hematopoietic cell transplantation: A single-center experience. Open forum infectious diseases 2018; 5(5). |

| Num | Author, Year & country | Overall Sample Size | Age-group | Population according to study setting | Older adults or risk group | Study Years | Data collection | Study design | Outcome reported | Full citation |
|---|---|---|---|---|---|---|---|---|---|---|
| 86 | Steensels, 2019, Belgium | 397 | ≥18 | Medically attended, immunocompromised | Immunodeficient (patients with any disease and/or treatment known to impair the immune system, such as solid organ transplant under immunosuppressive therapy, solid or hematological malignancy under chemotherapy, or other underlying disease needing long-term high-dose corticosteroid therapy or immunosuppressive therapy Patients infected with HIV with a CD4 count < 200/ mm3 were also included). | 2014-2015 | Continuous (annual) | Cohort | RSV proportion (HR) | Steensels D, Reynders M, Descheemaeker P, et al. Epidemiology and clinical impact of viral, atypical, and fungal respiratory pathogens in symptomatic immunocompromised patients: a two-center study using a multi-parameter customized respiratory Taqman array card. European Journal of Clinical Microbiology and Infectious Diseases 2019; 38(8): 1507-14. |
| 87 | Stolz, 2019, Switzerland | 445 | >40 | Community cohort, COPD | Cardiopulmonary, lung disease | 2011-2015 | Continuous (annual) | Trial | Cardiopulmonary complications proportion (HR); RSV proportion (HR); URTI proportion (HR) | Stolz D, Papakonstantinou E, Grize L, et al. Time-course of upper respiratory tract viral infection and COPD exacerbation. Eur Respir J 2019; 54(4). |
| 88 | Sundaram, 2014, USA | 992 | ≥50; ≥65 | Medically attended | Older adults, CKD, lung disease, cardiac disease | 2004-2010 | Seasonal | Cohort | RSV proportion (HR) | Sundaram ME, Meece JK, Sifakis F, Gasser RAJ, Belongia EA. Medically attended respiratory syncytial virus infections in adults aged ≥ 50 years: clinical characteristics and outcomes. Clinical infectious diseases: an official publication of the Infectious Diseases Society of America 2014; 58(3): 342-9. |
| 89 | Tanner, 2012, UK | 449 | ≥65 | Medically attended | Older adults | 2009-2010 | Seasonal | Surveillance | RSV proportion (Elderly) | Tanner H, Boxall E, Osman H. Respiratory viral infections during the 2009-2010 winter season in Central England, UK: Incidence and patterns of multiple virus co-infections. European Journal of Clinical Microbiology and Infectious Diseases 2012; 31(11): 3001-6. |

| Num | Author, Year & country | Overall Sample Size | Age-group | Population according to study setting | Older adults or risk group | Study Years | Data collection | Study design | Outcome reported | Full citation |
|---|---|---|---|---|---|---|---|---|---|---|
| 90 | Teh, 2015, Australia | 75 | ≥18 | medically attended, haematology patients | Immunodeficient (multiple myeloma patients) | 2009-2012 | Continuous (annual) | Retrospective case series | Case fatality rate (HR); Hospitalisation proportion (HR); ICU admission proportion (HR); LRTI proportion (HR); RSV proportion (HR); URTI proportion (HR) | Teh BW, Worth LJ, Harrison SJ, Thursky KA, Slavin MA. Risks and burden of viral respiratory tract infections in patients with multiple myeloma in the era of immunomodulatory drugs and bortezomib: experience at an Australian Cancer Hospital. Supportive Care in Cancer 2015; 23(7): 1901-6. |
| 91 | Theodoropoulos, 2013, USA | 249 | ≥18 | Medically attended, SOT patients | immunodeficient (solid organ transplant patients) | 2009-2012 | Continuous (annual) | Cohort | RSV proportion (HR) | Theodoropoulos N, Martin S, Ho B, Ison M. Non-influenza respiratory viral infections in solid organ transplant patients at two midwestern transplant centers, 2009-2012. American Journal of Transplantation 2013; 13: 214. |
| 92 | Thomas, 2019, USA | 27489 | ≥65 | Medically attended, influenza vaccinated | Older adults | 2010-2017 | Seasonal | Vaccine efficacy study | RSV proportion (Elderly) | Thomas Ray G, Lewis N, Klein NP, et al. Intraseason waning of influenza vaccine effectiveness. Clinical Infectious Diseases 2019; 68(10): 1623-30. |
| 93 | Tramuto, 2016, Italy | 75 | ≥65 | Medically attended, ILI, ICU inpatients | Older adults | 2009-2012 | Continuous (annual) | Cross-sectional | RSV proportion (Elderly) | Tramuto F, Maida CM, Napoli G, et al. Burden and viral aetiology of influenza-like illness and acute respiratory infection in intensive care units. Microbes and Infection 2016; 18(4): 270-6. |
| 94 | Van Beek, 2017, Netherlands | 3119 | ≥60 | Community cohort | Older adults | 2011-2013 | Seasonal | Cohort | RSV proportion (Elderly); RSV incidence (Elderly) | Van Beek J, Veenhoven RH, Bruin JP, et al. Influenza-like illness incidence is not reduced by influenza vaccination in a cohort of older adults, despite effectively reducing laboratory-confirmed influenza virus infections. Journal of Infectious Diseases 2017; 216(4): 415-24. |
| 95 | Varghese, 2018, Australia | 447 | ≥65 | Medically attended, ILI | Older adults | 2010-2013 | Continuous (annual) | Surveillance | RSV proportion (Elderly) | Varghese BM, Dent E, Chilver M, Cameron S, Stocks NP. Epidemiology of viral respiratory infections in Australian working-age adults (20-64 years): 2010-2013. Epidemiology and infection 2018; 146(5): 619-26. |
| 96 | Visseaux, 2017, France | 2449 | >60 | Medically attended | Older adults | 2011-2016 | Continuous (annual) | Surveillance | RSV proportion (Elderly) | Visseaux B, Burdet C, Voiriot G, et al. Prevalence of respiratory viruses among adults, by season, age, respiratory tract region and type of medical unit in Paris, France, from 2011 to 2016. PLoS ONE 2017; 12(7): e0180888. |

| Num | Author, Year & country | Overall Sample Size | Age-group | Population according to study setting | Older adults or risk group | Study Years | Data collection | Study design | Outcome reported | Full citation |
|---|---|---|---|---|---|---|---|---|---|---|
| 97 | Walker, 2014, USA | 502 | ≥18, ≥60 | Medically attended, inpatients | Older adults, CKD, diabetes, immunodeficient (patients with hematologic malignancy, solid tumour malignancy, autoimmune or rheumatologic disease, asplenia, primary immunodeficiency, or were the recipient of a solid organ transplant), lung disease, cardiac disease) | 2009-2010 | Continuous (annual) | Retrospective case series | RSV proportion (Elderly); RSV proportion (HR) | Walker E, Ison MG. Respiratory viral infections among hospitalized adults: Experience of a single tertiary healthcare hospital. Influenza and other respiratory viruses 2014; 8(3): 282-92. |
| 98 | Wansaula, 2016, USA | 156 | ≥18; >65 | Medically attended, SARI inpatients | Older adults, immunosuppression (reason not specified), lung disease, cardiac disease | 2010-2014 | Continuous (annual) | Surveillance | RSV proportion (Elderly); RSV proportion (HR) | Wansaula Z, Olsen SJ, Casal MG, et al. Surveillance for severe acute respiratory infections in Southern Arizona, 2010-2014. Influenza and other respiratory viruses 2016; 10(3): 161-9. |
| 99 | Weinberg, 2010, USA | 60 | ≥18 | Community cohort, SOT patients | Immunodeficient (lung transplant recipients) | 2005-2007 | Continuous (annual) | Cohort | Case fatality rate (HR); Hospitalisation proportion (HR); proportion (HR); RSV proportion (HR) | Weinberg A, Lyu DM, Li S, Marquesen J, Zamora MR. Incidence and morbidity of human metapneumovirus and other community-acquired respiratory viruses in lung transplant recipients. Transplant Infectious Disease 2010; 12(4): 330-5. |
| 100 | Widmer, 2012, USA | 508 | ≥65 | Medically attended, inpatients | Older adults, diabetes, immunodeficient (patients with transplants, cancer, splenectomy, HIV/AIDS, steroid use or chemotherapy, immunosuppression), institutionalised older adults, lung disease, cardiac disease | 2006-2009 | Seasonal | Vaccine efficacy study | RSV proportion (Elderly); RSV proportion (HR) | Widmer K, Zhu Y, Williams JV, Griffin MR, Edwards KM, Talbot HK. Rates of hospitalizations for respiratory syncytial virus, human metapneumovirus, and influenza virus in older adults. Journal of Infectious Diseases 2012; 206(1): 56-62. |

| Num | Author, Year & country | Overall Sample Size | Age-group | Population according to study setting | Older adults or risk group | Study Years | Data collection | Study design | Outcome reported | Full citation |
|---|---|---|---|---|---|---|---|---|---|---|
| 101 | Widmer, 2014, USA | 1248 | ≥18; ≥65 | Medically attended | Older adults, diabetes, immunodeficient (Transplant, cancer, splenectomy, HIV/AIDS, steroid use, chemotherapy, haemoglobinopathy, immunosuppression), institutionalised older adults, lung disease, cardiac disease | 2009-2010 | Continuous (annual) | Vaccine efficacy study | RSV proportion (HR) | Widmer K, Griffin MR, Zhu Y, Williams JV, Talbot HK. Respiratory syncytial virus- and human metapneumovirus-associated emergency department and hospital burden in adults. Influenza and other respiratory viruses 2014; 8(3): 347-52. |
| 102 | Yousaf, 2017, USA | 23 | ≥18 | Medically attended, RSV-positive inpatients | Cardiopulmonary | 2015-2017 | Continuous (annual) | Retrospective case series | Case fatality rate (HR) | Yousaf H, Ramage J. Severe respiratory syncytial virus (RSV) in the adult (dec 2015-jan 2017). Chest 2017; 152(4): A343. |
| 103 | Zambon, 2001, UK | 167 | ≥65 | Medically attended, ILI | Older adults | 1995-1998 | Seasonal | Surveillance | RSV proportion (Elderly) | Zambon MC, Stockton JD, Clewley JP, Fleming DM. Contribution of influenza and respiratory syncytial virus to community cases of influenza-like illness: An observational study. Lancet 2001; 358(9291): 1410-6. |

*HR= high-risk group; SS = systematic sampling; CI = sampling by clinical indication; ILI = influenza like illness; ARI = acute respiratory infection; PNM=pneumonia; IP = inpatients; ICU= intensive care unit; AECOPD = acute exacerbation of COPD; HSCT =Hematopoietic stem-cell transplantation; SOT= Solid organ transplant;*

**Supplementary Table 7.** RSV incidence in older adults (annual and seasonal studies).

| Study | Country | Data collection | Setting | Age Group | Sampling | Positive | Person-years | Incidence (/1000 person-years) [95% CI] |
|---|---|---|---|---|---|---|---|---|
| Huijts, 2018* | NL | Annual | Medically attended | ≥65 | SS PNM | 91 | 335449 | 0.27 [0.22, 0.33] |
| Belongia, 2018* | US | Seasonal | Medically attended | ≥60 | SS ARI | 243 | 32261 | 7.53 [6.62, 8.51] |
| Falsey, 2005 | US | Seasonal | Community cohort | ≥65 | SS ARI | 46 | 426 | 108 [79.07; 141.37] |
| Van Beek, 2017 | NL | Seasonal | Community cohort | ≥60 | SS ILI | 25 | 4723 | 5.29 [3.42, 7.56] |
| RE Model for seasonal data (Q=288.15, p=0.00; $I^2$= 99.1%) | | | | | | | | 16.11 (3.52,73.83) |

*REM= random-effect model, Q= Cochran's Q test, $I^2$ = $I^2$ statistic, SS= systematic sampling, CI = sampling by clinical indication, 95% CI = 95% confidence interval, ILI = influenza like illness, ARI = acute respiratory infection, PNM=pneumonia, IP = inpatients, Huijts, 2018: Capita trial population; no active community-based follow-up. Cases ascertained when they presented to healthcare facilities so classifying as medically attended. Belongia, 2018: Fever & cough included in eligibility criteria most seasons.*

**Supplementary Table 8.** Estimated proportion of symptomatic respiratory infection attributable to RSV and estimated RSV case fatality proportion among older adults and HR adults by geographical location.

| Region | Proportion | | | | CFP | |
|---|---|---|---|---|---|---|
| | Annual | | Seasonal | | | |
| | Obs | Proportion % (95% CI) | Obs | Proportion % (95% CI) | Obs | Proportion % (95% CI) |
| **Older adults** | | | | | | |
| **North America** | 4 | 4.49 [1.50-12.67] | 8 | 6.72 [4.78, 9.38] | | - |
| **Europe** | 12 | 5.09 [3.42-7.50] | 24 | 6.65 [4.79, 8.87] | | - |
| **Western Pacific** | 3 | 3.45 [2.10-5.61] | 0 | - | | - |
| **High-risk adults** | | | | | | |
| **North America** | 17 | 5.44 [3.60, 8.13] | 18 | 10.07 [8.05, 12.54] | 13 | 7.73 [4.18,13.88] |
| **Europe** | 15 | 11.21 [6.45, 18.78] | 18 | 6.22 [4.49, 8.55] | 14 | 13.00 [9.16, 18.12] |
| **Western Pacific** | 10 | 5.32 [3.17, 8.78] | 2 | - | 2 | - |

*Obs= Observations, CFP= case fatality proportion, 95% CI = 95% confidence interval.*

**Supplementary Table 9.** URTI, LRTI and bronchitis proportion among older adults and HR groups.

| Study | Country | Data collection | Population & age | Sampling | n | RSV+ | Total participants | Proportion [95% CI] | Group or risk group |
|---|---|---|---|---|---|---|---|---|---|
| **1. Older adults** | | | | | | | | | |
| ***URTI*** | | | | | | | | | |
| Van Beek, 2017 | NL | Seasonal | Community cohort; ≥60 | SS ILI | 25 | 25 | 4360 | 100.00 [86.68 – 100.00] | Older adults |
| **2. HR adults** | | | | | | | | | |
| ***URTI*** | | | | | | | | | |
| Stolz, 2019 | CH | Annual | Community cohort; >40 | SS ARI | 16 | 16 | 450 | 100.00 [80.64, 100] | Cardiopulmonary |
| Hopkins, 2008 | AU | Annual | Community cohort; ≥18 | SS ILI | 5 | 18 | 89 | 27.78 [12.50, 50.87] | Institutionalised older adults |
| Kumar, 2005 | CA | Annual | Medically attended; ≥18, OP | SS RVI | 1 | 6 | 6 | 100 [60.97, 100] | Immunodeficiency |
| Damlaj, 2016 | US | Annual | Medically attended; ≥18, RSV+ | CI | 3 | 35 | 45 | 77.78 [63.73, 87.46] | Immunodeficiency |
| Spahr, 2018 | CH | Annual | Medically attended; ≥18, RSV+ | CI ARI | 4 | 22 | 33 | 66.67 [49.61, 80.25] | Immunodeficiency |
| Teh, 2015 | AU | Annual | Medically attended; ≥18, | CI RVI | 3 | 10 | 15 | 66.67 [41.71, 84.82] | Immunodeficiency |
| Garcia-Noblejas, 2015 | ES | Annual | Medically attended; ≥18, | CI ARI | 5 | 21 | 32 | 65.62 [48.31, 79.59] | Immunodeficiency |
| Pinana, 2017 | ES | Annual | Medically attended; ≥18, RSV+ | CI | 1 | 15 | 23 | 65.22 [44.89, 81.19] | Immunodeficiency |
| Khanna, 2018 | CH | Annual | Medically attended; ≥18, RSV+ | SS ARI | 4 | 22 | 34 | 64.71 [47.91, 78.51] | Immunodeficiency |
| Slade, 2017* | US | Annual | Medically attended; ≥18, | SS ARI | 3 | 3 | 5 | 60 [23.07, 88.24] | Immunodeficiency |
| Chatzis, 2018 | CH | Annual | Medically attended; ≥18, RSV+ | CI ARI | 2 | 90 | 175 | 51.43 [44.07, 58.72] | Immunodeficiency |
| Gueller, 2013* | DE | Seasonal | Medically attended; ≥18, IP | SS | 4 | 5 | 10 | 50 [23.66, 76.34] | Immunodeficiency |
| Sanghavi, 2012 | US | Seasonal | Medically attended; ≥18, | CI ARI | 5 | 8 | 17 | 47.06 [26.17, 69.04] | Immunodeficiency |
| Li, 2012* | US | Annual | Medically attended; ≥18, RSV+ | CI | 2 | 9 | 21 | 42.86 [24.47, 63.45] | Immunodeficiency |
| Pilie, 2015 | US | Annual | Medically attended; ≥18, RSV+ IP | CI | 3 | 21 | 69 | 30.43 [20.85, 42.08] | Immunodeficiency |
| Roghmann, 2003 | US | Seasonal | Community cohort; ≥18, | SS ARI | 2 | 6 | 11 | 54.55 [28.01, 78.73] | Immunodeficiency |
| Peghin, 2017 | ES | Annual | Community cohort; ≥18, | SS ARI | 0 | 2 | 7 | 28.57 [8.22, 64.11] | Immunodeficiency |
| *URTI: REM for Immunodeficiency (Q=37.24, p=0.00, $I^2$=61.9%)* | | | | | | | | *56.80 [48.13, 65.07]* | Immunodeficiency |
| ***LRTI*** | | | | | | | | | |
| D'Angelo, 2016* | US | Annual | Medically attended; ≥50 | SS ARI | 3 | 5 | 6 | 83.33 [43.65, 96.99] | Immunodeficiency |
| Pilie, 2015 | US | Annual | Medically attended; ≥18, RSV+ IP | CI | 3 | 48 | 69 | 69.57 [57.92, 79.15] | Immunodeficiency |
| Li, 2012* | US | Annual | Medically attended; ≥18, RSV+ | CI | 2 | 12 | 21 | 57.14 [36.55, 75.53] | Immunodeficiency |
| Sanghavi, 2012 | US | Seasonal | Medically attended; ≥18 | CI ARI | 5 | 9 | 17 | 52.94 [30.96, 73.83] | Immunodeficiency |
| Gueller, 2013* | DE | Seasonal | Medically attended; ≥18, IP | SS | 4 | 5 | 10 | 50 [23.66, 76.34] | Immunodeficiency |

| Study | Country | Data collection | Population & age | Sampling | n | RSV+ | Total participants | Proportion [95% CI] | Group or risk group |
|---|---|---|---|---|---|---|---|---|---|
| Chatzis, 2018 | CH | Annual | Medically attended; ≥18, RSV+ | CI ARI | 2 | 85 | 175 | 48.57 [41.28, 55.93] | Immunodeficiency |
| Damlaj, 2016 | US | Annual | Medically attended; ≥18, RSV+ | CI | 3 | 21 | 45 | 46.67 [32.94, 60.92] | Immunodeficiency |
| Slade, 2017* | US | Annual | Medically attended; ≥18 | SS ARI | 3 | 2 | 5 | 40 [11.76, 76.93] | Immunodeficiency |
| Khanna, 2018 | CH | Annual | Medically attended; ≥18, RSV+ | SS ARI | 4 | 12 | 34 | 35.29 [21.49, 52.09] | Immunodeficiency |
| Pinana, 2017 | ES | Annual | Medically attended; ≥18, RSV+ | CI | 1 | 8 | 23 | 34.78 [18.81, 55.11] | Immunodeficiency |
| Garcia-Noblejas, 2015 | ES | Annual | Medically attended; ≥18 | CI ARI | 5 | 11 | 32 | 34.38 [20.41, 51.69] | Immunodeficiency |
| Teh, 2015 | AU | Annual | Medically attended; ≥18 | CI RVI | 3 | 5 | 15 | 33.33 [15.18, 58.29] | Immunodeficiency |
| Spahr, 2018 | CH | Annual | Medically attended; ≥18, RSV+ | CI ARI | 4 | 9 | 33 | 27.27 [15.07, 44.22] | Immunodeficiency |
| Kumar, 2005 | CA | Annual | Medically attended; ≥18, OP | SS RVI | 1 | 0 | 6 | 0 [0, 39.03] | Immunodeficiency |
| Peghin, 2017 | ES | Annual | Community cohort; ≥18 | SS ARI | 0 | 5 | 7 | 71.43 [35.89, 91.78] | Immunodeficiency |
| Roghmann, 2003 | US | Seasonal | Community cohort; ≥18 | SS ARI | 2 | 3 | 11 | 27.27 [9.75, 56.56] | Immunodeficiency |
| Milstone, 2006 | US | Seasonal | Community cohort; ≥18 | SS ARI | 1 | 2 | 8 | 25 [7.15, 59.07] | Immunodeficiency |
| *LRTI: REM for Immunodeficiency (Q=35.48, p=0.00, $I^2$=54.9%)* | | | | | | | | *44.53 [36.83, 52.49]* | Immunodeficiency |
| ***Bronchitis*** | | | | | | | | | |
| Peghin, 2017 | ES | Annual | Community cohort; ≥18 | SS ARI | 2 | 7 | 98 | 28.57 [8.22, 64.11] | Immunodeficiency |
| Gueller, 2013 | DE | Seasonal | IP; ≥18 | SS | 9 | 10 | 29 | 90.00 [59.58, 98.21] | Immunodeficiency |

*REM= random-effect model, Q= Cochran's Q test, $I^2$ = $I^2$ statistic, SS = systematic sampling, CI = sampling by clinical indication, 95% CI = 95% confidence interval, ARI = acute respiratory infection, ILI = influenza like illness, AEC = acute exacerbation of COPD, RVI = respiratory virus infection.*

**Supplementary Table 10.** RSV signs and symptoms description in older and HR adults. The table shows mean, min, and max number of percentages of patients reporting signs and symptoms and the amount of studies (#Studies column) and specific studies identified (Citation) informing on RSV signs and symptoms percentages. Signs and symptoms affecting >50% of patients are in red. #Patients column shows the total number of patients reporting on a specific sign or symptom from all included studies from #Studies column.

| Signs & symptoms | #Studies (citations) | Citation | #Patients | RSV cases with symptom (%) | | | |
|---|---|---|---|---|---|---|---|
| | | | | Median | Mean | Min | Max |
| **1.Older adults** | | | | | | | |
| ***1.1. Self-reported symptoms*** | | | | | | | |
| Cough | 6 | Volling, 2014; Saez-Lopez, 2019; Puig-Barbera, 2012; Lee, 2019; Lee, 2011; Belongia, 2018 | 407 | 86.0 | 81.5 | 44.9 | 97.8 |
| Weakness/Malaise | 3 | Volling, 2014; Saez-Lopez, 2019; Puig-Barbera, 2012 | 131 | 86.7 | 75.8 | 50.0 | 90.7 |
| Shortness of breath | 6 | Volling, 2014; Saez-Lopez, 2019; Puig-Barbera, 2012; Lee, 2019; Lee, 2011; Belongia, 2018 | 309 | 72.3 | 67.7 | 19.3 | 94.0 |
| Sputum | 4 | Volling, 2014; Lee, 2019; Lee, 2011; Belongia, 2018 | 260 | 56.1 | 59.3 | 39.1 | 86.0 |
| Fever | 5 | Volling, 2014; Saez-Lopez, 2019; Puig-Barbera, 2012; Lee, 2019; Belongia, 2018 | 252 | 53.3 | 53.4 | 34.6 | 74.1 |
| Sore throat | 5 | Volling, 2014; Saez-Lopez, 2019; Puig-Barbera, 2012; Lee, 2019; Belongia, 2018 | 270 | 57.4 | 49.0 | 18.6 | 71.1 |
| Wheezing | 4 | Volling, 2014; Lee, 2019; Lee, 2011; Belongia, 2018 | 169 | 45.1 | 46.6 | 16.1 | 80.0 |
| Runny nose | 1 | Lee, 2019 | 46 | 41.8 | 41.8 | 41.8 | 41.8 |
| Myalgia | 3 | Saez-Lopez, 2019; Lee, 2019; Belongia, 2018 | 107 | 23.6 | 40.7 | 18.5 | 80.0 |
| Headache | 2 | Saez-Lopez, 2019; Lee, 2019 | 47 | 39.8 | 39.8 | 17.3 | 62.2 |
| Hoarseness | 1 | Lee, 2019 | 43 | 39.1 | 39.1 | 39.1 | 39.1 |
| Productive cough | 2 | Volling, 2014; Belongia, 2018 | 137 | 35.7 | 35.7 | 23.3 | 48.2 |
| Congested nose | 2 | Volling, 2014; Lee, 2019 | 65 | 32.0 | 32.0 | 22.1 | 41.8 |
| Fatigue | 1 | Lee, 2019 | 33 | 30.0 | 30.0 | 30.0 | 30.0 |
| Vomiting | 2 | Volling, 2014; Lee, 2019 | 28 | 14.5 | 14.5 | 12.7 | 16.3 |
| Chest pain | 3 | Volling, 2014; Lee, 2019; Belongia, 2018 | 40 | 7.4 | 10.4 | 6.4 | 17.4 |
| Sweating | 1 | Lee, 2019 | 6 | 5.5 | 5.5 | 5.5 | 5.5 |
| Haemoptysis | 1 | Belongia, 2018 | 3 | 5.5 | 1.2 | 1.2 | 1.2 |
| ***1.2. Signs on examination*** | | | | | | | |
| Wheezing | 1 | Belongia, 2018 | 49 | 20.2 | 20.2 | 20.2 | 20.2 |
| Rhonchi | 1 | Belongia, 2018 | 31 | 12.8 | 12.8 | 12.8 | 12.8 |
| Crackles | 1 | Belongia, 2018 | 23 | 9.5 | 9.5 | 9.5 | 9.5 |
| Fever | 1 | Belongia, 2018 | 20 | 8.2 | 8.2 | 8.2 | 8.2 |
| Reduced breath sounds | 1 | Belongia, 2018 | 19 | 7.8 | 7.8 | 7.8 | 7.8 |
| Tachypnoea | 1 | Belongia, 2018 | 16 | 6.6 | 6.6 | 6.6 | 6.6 |
| Reduced oxygen saturation | 1 | Belongia, 2018 | 11 | 4.5 | 4.5 | 4.5 | 4.5 |
| Respiratory Distress | 1 | Belongia, 2018 | 6 | 2.5 | 2.5 | 2.5 | 2.5 |
| **2. HR adults: patients with cardiopulmonary disease** | | | | | | | |
| ***2.1 Self-reported symptoms*** | | | | | | | |
| Cough | 2 | Walsh, 2007; Camargo, 2008 | 121 | 98.7 | 98.7 | 97.5 | 100.0 |
| Shortness of breath | 2 | Walsh, 2007; Camargo, 2008 | 118 | 97.5 | 97.5 | 94.9 | 100.0 |

| Signs & symptoms | #Studies (citations) | Citation | #Patients | RSV cases with symptom (%) | | | |
|---|---|---|---|---|---|---|---|
| | | | | Median | Mean | Min | Max |
| Sputum | 1 | Camargo, 2008 | 5 | 83.3 | 83.3 | 83.3 | 83.3 |
| Nasal congestion | 2 | Walsh, 2007; Camargo, 2008 | 85 | 75.6 | 75.6 | 67.8 | 83.3 |
| Wheezing | 1 | Walsh, 2007 | 86 | 72.9 | 72.9 | 72.9 | 72.9 |
| Discoloured sputum | 1 | Camargo, 2008 | 4 | 66.7 | 66.7 | 66.7 | 66.7 |
| Fever | 2 | Walsh, 2007; Camargo, 2008 | 67 | 52.1 | 52.1 | 50.0 | 54.2 |
| ConstSympt | 1 | Walsh, 2007 | 47 | 39.8 | 39.8 | 39.8 | 39.8 |
| Hoarseness | 1 | Walsh, 2007 | 33 | 28.0 | 28.0 | 28.0 | 28.0 |
| Sore throat | 2 | Walsh, 2007; Camargo, 2008 | 35 | 22.7 | 22.7 | 16.7 | 28.8 |
| Chest pain | 1 | Walsh, 2007 | 22 | 18.6 | 18.6 | 18.6 | 18.6 |
| Runny nose | 1 | Walsh, 2007 | 13 | 11.0 | 11.0 | 11.0 | 11.0 |
| ***2.2. Signs on examinations*** | | | | | | | |
| Wheezing | 1 | Walsh, 2007 | 97 | 82.2 | 82.2 | 82.2 | 82.2 |
| Crackles | 1 | Walsh, 2007 | 74 | 62.7 | 62.7 | 62.7 | 62.7 |
| Fever | 1 | Walsh, 2007 | 15 | 12.7 | 12.7 | 12.7 | 12.7 |
| **3. HR adults: immunodeficient patients** | | | | | | | |
| ***3.1 Self-reported symptoms*** | | | | | | | |
| Cough | 3 | Lee, 2019; Gueller, 2013; Garcia-Noblejas, 2015 | 93 | 72.2 | 71.6 | 62.5 | 80.0 |
| Wheezing | 1 | Lee, 2019 | 55 | 61.1 | 61.1 | 61.1 | 61.1 |
| Sputum | 1 | Lee, 2019 | 47 | 52.2 | 52.2 | 52.2 | 52.2 |
| Nasal congestion | 2 | Lee, 2019; Gueller, 2013 | 52 | 46.7 | 46.7 | 40.0 | 53.3 |
| Fatigue | 1 | Lee, 2019 | 39 | 43.3 | 43.3 | 43.3 | 43.3 |
| Runny nose | 2 | Lee, 2019; Garcia-Noblejas, 2015 | 56 | 42.2 | 42.2 | 34.4 | 50.0 |
| Fever | 5 | Pinana, 2017; Pilie, 2015; Mikulsa, 2014; Lee, 2019; Garcia-Noblejas, 2015 | 114 | 34.4 | 38.0 | 16.7 | 66.7 |
| Myalgia | 1 | Lee, 2019 | 34 | 37.8 | 37.8 | 37.8 | 37.8 |
| Shortness of breath | 3 | Lee, 2019; Gueller, 2013; Garcia-Noblejas, 2015; | 73 | 12.5 | 32.7 | 10.0 | 75.6 |
| Sore throat | 1 | Lee, 2019 | 28 | 31.1 | 31.1 | 31.1 | 31.1 |
| Hoarseness | 1 | Lee, 2019 | 23 | 25.6 | 25.6 | 25.6 | 25.6 |
| Headache | 1 | Lee, 2019 | 21 | 23.3 | 23.3 | 23.3 | 23.3 |
| Sweating | 1 | Lee, 2019 | 11 | 12.2 | 12.2 | 12.2 | 12.2 |
| Odynophagia | 1 | Garcia-Noblejas, 2015 | 3 | 9.4 | 9.4 | 9.4 | 9.4 |
| Vomiting | 1 | Lee, 2019 | 8 | 8.9 | 8.9 | 8.9 | 8.9 |
| Chest Pain | 1 | Lee, 2019 | 6 | 6.7 | 6.7 | 6.7 | 6.7 |
| ***3.2 Signs on examinations*** | | | | | | | |
| Fever | 2 | Pinana, 2017; Pilie, 2015 | 40 | 36.2 | 36.2 | 21.7 | 50.7 |
| **4. HR adults: institutionalised patients** | | | | | | | |
| ***4.1 Self-reported symptoms*** | | | | | | | |
| Cough | 1 | Hequet, 2019 | 34 | 89.5 | 89.5 | 89.5 | 89.5 |
| Weakness/Malaise | 1 | Hequet, 2019 | 28 | 73.7 | 73.7 | 73.7 | 73.7 |
| Fever | 1 | Hequet, 2019 | 27 | 71.1 | 71.1 | 71.1 | 71.1 |

**Supplementary Table 11.A.** RSV severe outcomes in older adults.

| Study | Country | Data collection | Population & age | Sampling | n | N | Total participants | Proportion [95% CI] |
|---|---|---|---|---|---|---|---|---|
| ***Pneumonia*** | | | | | | | | |
| Ansaldi, 2012 | IT | Seasonal | Community cohort; ≥60 | SS ILI | 0 | 2 | 45 | 0.00 [0.00, 65.76] |
| Jeannoel, 2019 | FR | Seasonal | Medically attended; ≥65 | CI | 56 | 165 | 165 | 33.94 [27.15, 41.46] |
| Puig-Barbera, 2012 | ES | Seasonal | Medically attended; ≥60 | SS ILI | 14 | 54 | 799 | 25.93 [16.12, 38.93] |
| Aronen, 2019 | FI | Annual | Medically attended; ≥65 | SS ARI | 3 | 22 | 382 | 13.64 [4.75, 33.33] |
| *Pneumonia: REM for older adults (Q=4.41, p=0.22, $I^2$=40.4%)* | | | | | | | | *27.44 [18.74, 38.29]* |
| ***Respiratory failure*** | | | | | | | | |
| Jeannoel, 2019 | FR | Seasonal | Medically attended, RSV+ ARI; ≥65 | CI | 33 | 165 | 165 | 20.00 [14.61, 26.75] |
| ***ARDS*** | | | | | | | | |
| Jeannoel, 2019 | FR | Seasonal | Medically attended, RSV+ ARI; ≥65 | CI | 33 | 165 | 165 | 20.00 [14.61, 26.75] |
| ***Cardiopulmonary complications*** | | | | | | | | |
| Belongia et al, 2018 | US | Seasonal | Medically attended; ≥60 | SS ARI/ILI | 27 | 243 | 2257 | 11.11 [7.75, 15.68] |
| ***Hospitalisation*** | | | | | | | | |
| Charles, 2008 | AU | Annual | Medically attended; ≥65 | SS PNM | 13 | 13 | 587 | 100 [77.19, 100] |
| Belongia, 2018 | US | Seasonal | Medically attended; ≥60 | SS ARI | 29 | 243 | 2257 | 11.93 [8.44, 16.61] |
| Falsey, 2005 | US | Seasonal | Community cohort; ≥65 | SS ARI | 0 | 46 | 608 | 0.00 [0.00, 7.71] |
| *Hospitalisation of older adults: REM for older adults (Q=16.63, p=0.00, $I^2$=92.7%)* | | | | | | | | *24.48 [0.43, 96.07]* |
| ***Admissions to ICU*** | | | | | | | | |
| Jeannoel, 2019 | FR | Seasonal | Medically attended; ≥65 | CI | 41 | 165 | 165 | 24.85 [18.88, 31.96] |
| Belongia, 2018 | US | Seasonal | Medically attended; ≥60 | SS ARI | 0 | 29 | 2257 | 0.00 [0.00, 11.7] |
| Puig-Barbera, 2012 | ES | Seasonal | Medically attended; ≥60 | SS ILI | 0 | 54 | 799 | 0.00 [0.00, 6.64] |
| *ICU admission of older adults: REM for older adults (Q=10.38, p=0.01, $I^2$=76.8%)* | | | | | | | | *5.01 [0.47, 37.36]* |
| ***Ventilatory support*** | | | | | | | | |
| Belongia, 2018 | US | Seasonal | Medically attended; ≥60 | SS ARI | 0 | 29 | 2257 | 0.00 [0.00, 11.70] |
| Charles, 2008 | AU | Annual | Medically attended; ≥65 | SS PNM | 0 | 13 | 587 | 0.00 [0.00, 22.81] |

*REM= random-effect model, Q= Cochran's Q test, $I^2$ = $I^2$ statistic, SS = systematic sampling, CI = sampling by clinical indication, 95% CI = 95% confidence interval, ILI = influenza like illness, ARI = acute respiratory infection, PNM: pneumonia.*

**Supplementary Table 11.B.** RSV severe outcomes in HR adults.

| Study | Country | Data collection | Population & age | Sampling | n | N | Total participants | Proportion [95% CI] | Risk group |
|---|---|---|---|---|---|---|---|---|---|
| ***Pneumonia*** | | | | | | | | | |
| Falsey, 2005 | US | Seasonal | Medically attended, IP; ≥65 | SS ARI | 41 | 142 | 1388 | 28.87 [22.05, 36.81] | Cardiopulmonary |
| Chatzis, 2018 | US | Seasonal | Medically attended, ≥18, RSV+ | CI ARI | 2 | 62 | 175 | 35.43 [28.72, 42.76] | Immunodeficient |
| Gueller, 2013 | CH | Annual | Medically attended, ≥18, IP | SS | 4 | 1 | 10 | 10 [1.79, 40.42] | Immunodeficient |
| Kumar | DE | Seasonal | Medically attended, ≥18, OP | SS RVI | 1 | 0 | 6 | 0.00 [0.00, 39.03] | Immunodeficient |
| Milstone, 2006 | CA | Annual | Community cohort; ≥18, | SS ARI | 1 | 0 | 8 | 0.00 [0.00, 32.44] | Immunodeficient |
| Peghin, 2017 | US | Seasonal | Community cohort; ≥18, | SS ARI | 0 | 3 | 7 | 42.86 [15.82, 74.95] | Immunodeficient |
| Pile | ES | Annual | Medically attended, ≥18, RSV+ IP | CI | 3 | 27 | 69 | 39.13 [28.48, 50.93] | Immunodeficient |
| *Pneumonia: REM for immunodeficient (Q=7.02, p=0.22, $I^2$=0.0%)* | | | | | | | | *35.33 [29.78, 41.30]* | *Immunodeficiency* |
| *Pneumonia: REM for all risk groups (Q=7.02, p=0.22, $I^2$= 0.0%)* | | | | | | | | *33.00 [27.99, 38.43]* | *All risk-groups combined* |
| ***Respiratory failure*** | | | | | | | | | |
| Hopkins, 2008 | AU | Annual | Community cohort; ≥18 | SS ILI | 2 | 18 | | 11.11 [3.1, 32.8] | Institutionalised older adults |
| Renaud, 2013 | US | Annual | Medically attended, RSV+ ≥18 | CI | 16 | 23 | | 69.57 [49.13, 84.4] | Immunodeficiency |
| Khanna, 2018 | CH | Annual | Medically attended, RSV+ ≥18 | SS ARI | 3 | 34 | | 8.82 [3.05, 22.96] | Immunodeficiency |
| Roghmann, 2003 | US | Seasonal | Medically attended, ≥18 | SS ARI | 0 | 11 | | 0.00 [0.00, 25.88] | Immunodeficiency |
| *Respiratory failure: REM for immunodeficient (Q=21.11, p=0.00, $I^2$=89.1%)* | | | | | | | | *20.62 [2.22, 74.82]* | *Immunodeficiency* |
| *Respiratory failure: REM for all risk groups (Q=24.85, p= 0.00, $I^2$= 84.8 %)* | | | | | | | | *18.40 [3.65, 57.31]* | *All risk-groups combined* |
| ***ARDS*** | | | | | | | | | |
| Jeannoel, 2019 | FR | Seasonal | RSV+ ARI ≥18 | CI | 9 | 55 | 14792 | 16.36 [8.86, 28.26] | Cardiopulmonary |
| Jeannoel, 2019 | FR | Seasonal | RSV+ ARI ≥18 | CI | 5 | 16 | 14792 | 31.25 [14.16, 55.6] | Diabetes |
| Jeannoel, 2019 | FR | Seasonal | RSV+ ARI ≥18 | CI | 9 | 37 | 14792 | 24.32 [13.36, 40.12] | Immunodeficient |
| *ARDS: REM for all risk groups (Q=1.90, p= 0.39, $I^2$= 0.0%)* | | | | | | | | 21.79 [14.89, 30.72] | *All risk-groups combined* |
| ***Cardiopulmonary complications*** | | | | | | | | | |
| Stolz, 2019 | CH | Annual | Community cohort; >40 | SS ARI | 6 | 16 | 450 | 37.5 [18.48, 61.36] | Cardiopulmonary |
| ***ICU admissions*** | | | | | | | | | |
| Jeannoel, 2019 | FR | Seasonal | RSV+ ARI; ≥18 | CI | 16 | 55 | 14792 | 29.09 [18.77, 42.14] | Cardiopulmonary |
| Schmidt, 2019 | US | Annual | RSV+ IP; ≥18 | CI | 30 | 107 | 489 | 28.04 [20.40, 37.20] | Chronic Kidney Disease |
| Schmidt, 2019 | US | Annual | RSV+ IP; ≥18 | CI | 40 | 133 | 489 | 30.08 [22.93, 38.34] | Diabetes |
| Jeannoel, 2019 | FR | Seasonal | RSV+ ARI; ≥18 | CI | 8 | 16 | 14792 | 50.00 [28.00, 72.00] | Diabetes |
| Chatzis, 2018 | CH | Annual | Medically attended; ≥18; RSV+ | CI ARI | 2 | 17 | 58 | 29.31 [19.18, 42.01] | Immunodeficient |
| Damlaj, 2016 | US | Annual | Medically attended; ≥18; RSV+ | CI | 3 | 9 | 27 | 33.33 [18.64, 52.18] | Immunodeficient |
| Gorcea, 2015 | GB | Annual | Medically attended; ≥18; RSV+ | CI | 3 | 6 | 20 | 30 [14.55, 51.9] | Immunodeficient |

| Study | Country | Data collection | Population & age | Sampling | n | N | Total participants | Proportion [95% CI] | Risk group |
|---|---|---|---|---|---|---|---|---|---|
| Jeannoel, 2019 | FR | Seasonal | Medically attended; ≥18; RSV+ ARI | CI | 4 | 13 | 37 | 35.14 [21.83, 51.24] | Immunodeficient |
| Khanna, 2018 | CH | Annual | Medically attended; ≥18; RSV+ | SS ARI | 4 | 5 | 16 | 31.25 [14.16, 55.6] | Immunodeficient |
| Peghin, 2017 | ES | Annual | Community cohort; ≥18 | SS ARI | 0 | 2 | 3 | 66.67 [20.77, 93.85] | Immunodeficient |
| Pilie, 2015 | US | Annual | Medically attended; ≥18; RSV+ IP | CI | 3 | 11 | 69 | 15.94 [9.14, 26.33] | Immunodeficient |
| Schmidt, 2019* | US | Annual | Medically attended; ≥18; RSV+ IP | CI | 3 | 18 | 216 | 8.33 [5.34, 12.79] | Immunodeficient |
| Spahr, 2018 | CH | Annual | Medically attended; ≥18; RSV+ | CI ARI | 4 | 2 | 10 | 20 [5.67, 50.98] | Immunodeficient |
| Teh, 2015 | AU | Annual | Medically attended; ≥18; | CI RVI | 3 | 1 | 6 | 16.67 [3.01, 56.35] | Immunodeficient |
| *ICU admission: REM for immunodeficient (Q=33.82, p=0.00, $I^2$=67.5%)* | | | | | | | | *24.09 [16.35, 34.01]* | *Immunodeficient* |
| *ICU admission: REM for all risk groups (Q=45.05, p=0.00, $I^2$=69.95%)* | | | | | | | | *26.74 [20.40, 34.22]* | *All risk-groups combined* |
| ***Ventilatory support*** | | | | | | | | | |
| Schmidt, 2019 | US | Annual | RSV+ IP ≥18 | CI | 12 | 107 | 489 | 11.21 [6.53, 18.59] | Chronic Kidney Disease |
| Schmidt, 2019 | US | Annual | RSV+ IP ≥18 | CI | 2 | 133 | 489 | 1.5 [0.41, 5.32] | Diabetes |
| Khanna, 2018 | CH | Annual | Medically attended; ≥18; RSV+ | SS ARI | 4 | 4 | 16 | 25 [10.18, 49.5] | Immunodeficient |
| Chatzis, 2018 | CH | Annual | Medically attended; ≥18; RSV+ | CI ARI | 2 | 13 | 58 | 22.41 [13.59, 34.66] | Immunodeficient |
| Pilie, 2015 | US | Annual | Medically attended; ≥18; RSV+ IP | CI | 3 | 7 | 69 | 10.14 [5, 19.49] | Immunodeficient |
| Spahr, 2018 | CH | Annual | Medically attended; ≥18; RSV+ | CI ARI | 4 | 1 | 10 | 10 [1.79, 40.42] | Immunodeficient |
| Schmidt, 2019* | US | Annual | Medically attended; ≥18; RSV+ IP | CI | 3 | 17 | 216 | 7.87 [4.97, 12.24] | Immunodeficient |
| *Ventilatory support: REM for immunodeficient (Q=33.82, p=0.00, $I^2$=63.7%)* | | | | | | | | *13.65 [7.87, 22.63]* | *Immunodeficient* |
| *Ventilatory support: REM for all risk groups (Q=21.01, p=0.00, $I^2$=77.0%)* | | | | | | | | *10.68 [5.87, 18.65]* | *All risk-groups combined* |
| ***Hospitalisation*** | | | | | | | | | |
| Hequet, 2019 | CH | Seasonal | Community cohort; ≥18 | SS ILI | 1 | 3 | 38 | 7.89 [2.72, 20.8] | Institutionalised older adults |
| Belongia, 2018 | US | Seasonal | Medically attended; ≥60 | SS ARI | 2 | 9 | 51 | 17.65 [9.57, 30.25] | Diabetes |
| Camargo, 2008 | US | Seasonal | Medically attended; ≥50 | SS AEC | 2 | 5 | 6 | 83.33 [43.65, 96.99] | Cardiopulmonary |
| Falsey, 2005 | US | Seasonal | Community cohort; ≥21 | SS ARI | 2 | 9 | 56 | 16.07 [8.69, 27.81] | Cardiopulmonary |
| Falsey, 2006 | US | Annual | Community cohort; ≥40 | SS ARI | 0 | 1 | 11 | 9.09 [1.62, 37.74] | Cardiopulmonary |
| Saraya, 2017 | JP | Annual | Medically attended; ≥18 | SS AEA | 0 | 3 | 3 | 100 [43.85, 100] | Asthma |
| D'Angelo, 2016 | US | Annual | Medically attended; ≥50 | SS ARI | 3 | 5 | 6 | 83.33 [43.65, 96.99] | Immunodeficiency |
| Damlaj, 2016 | US | Annual | Medically attended; ≥18 | CI | 3 | 27 | 45 | 60 [45.45, 72.98] | Immunodeficiency |
| Gorcea, 2015 | GB | Annual | Medically attended; ≥18 | CI | 3 | 20 | 35 | 57.14 [40.86, 72.02] | Immunodeficiency |
| Khanna, 2018 | CH | Annual | Medically attended; ≥18 | SS ARI | 4 | 16 | 34 | 47.06 [31.45, 63.26] | Immunodeficiency |
| Teh, 2015 | AU | Annual | Medically attended; ≥18 | CI RVI | 3 | 6 | 15 | 40 [19.82, 64.25] | Immunodeficiency |
| Belongia, 2018 | US | Seasonal | Medically attended; ≥60 | SS ARI | 2 | 5 | 15 | 33.33 [15.18, 58.29] | Immunodeficiency |
| Chatzis, 2018 | CH | Annual | Medically attended; ≥18 | CI ARI | 2 | 58 | 175 | 33.14 [26.6, 40.41] | Immunodeficiency |
| Spahr, 2018 | CH | Annual | Medically attended; ≥18 | CI ARI | 4 | 10 | 33 | 30.3 [17.38, 47.34] | Immunodeficiency |

| Study | Country | Data collection | Population & age | Sampling | n | N | Total participants | Proportion [95% CI] | Risk group |
|---|---|---|---|---|---|---|---|---|---|
| Pinana, 2017 | ES | Annual | Medically attended; ≥18 | CI | 1 | 2 | 23 | 8.7 [2.42, 26.8] | Immunodeficiency |
| Peghin, 2017 | ES | Annual | Community cohort; ≥18 | SS ARI | 0 | 3 | 7 | 42.86 [15.82, 74.95] | Immunodeficiency |
| Roghmann, 2003 | US | Seasonal | Community cohort; ≥18 | SS ARI | 2 | 3 | 11 | 27.27 [9.75, 56.56] | Immunodeficiency |
| Weinberg, 2010 | US | Annual | Community cohort; ≥18 | SS ARI | 0 | 3 | 13 | 23.08 [8.18, 50.26] | Immunodeficiency |
| Milstone, 2006 | US | Seasonal | Community cohort; ≥18 | SS ARI | 1 | 1 | 8 | 12.5 [2.24, 47.09] | Immunodeficiency |
| *Hospitalisation: REM for immunodeficient (Q=31.25, p=0.00, $I^2$=62%)* | | | | | | | | *38.30 [29.26, 48.23]* | *Immunodeficiency* |
| *Hospitalisation: REM for all risk groups (Q=66.89, p=0.00, $I^2$=77.7%)* | | | | | | | | *32.82[23.49, 43.74]* | *All risk-groups combined* |

*REM= random-effect model, Q= Cochran's Q test, $I^2$ = $I^2$ statistic, SS = systematic sampling, CI = sampling by clinical indication, 95% CI = 95% confidence interval, ILI = influenza like illness; ARI = acute respiratory infection, ARDS= acute respiratory distress syndrome, URTI=upper respiratory tract infection, LRTI=lower respiratory tract infection, AEC = acute exacerbation of COPD, RVI=respiratory virus infection, IP = inpatients, OP = outpatients, ED = Emergency department, ICU MV = intensive care unit, mechanically ventilated.*

**Supplementary Table 12.** RSV incidence in HR adults (annual and seasonal studies).

| Study | Data collection | Risk Group | Setting | Country | Age Group | Sampling | Positive | Person-years | Incidence (/1000 person-year) [95% CI] |
|---|---|---|---|---|---|---|---|---|---|
| Slade, 2017* | Annual | Immunodeficiency | Medically attended | US | ≥18 | SS ARI | 5 | 62 | 80.55 [26.15, 164.99] |
| Mahan, 2017* | Annual | Immunodeficiency | Medically attended | US | ≥18 | CI ARI | 3 | 66 | 45.45 [9.37, 109.47] |
| Peghin, 2017 | Annual | Immunodeficiency | Community cohort | ES | ≥18 | SS ARI | 7 | 333 | 21.01 [8.45, 39.19] |
| *Overall estimate for immunodeficient (annual) (Q=5.4, $I^2$ = 43.3%; p=0.07)* | | | | | | | | | *36.88 (17.82, 76.33)* |
| Stolz, 2019 | Annual | Cardiopulmonary | Community cohort | CH | >40 | SS ARI | 16 | 1013 | 15.8 [9.03, 24.43] |
| Chasqueira, 2018 | Seasonal | Institutionalised older adults | Community cohort | PT | ≥59 | SS ARI | 5 | 511 | 9.78 [3.18, 20.04] |
| Roghmann, 2003 | Seasonal | Immunodeficiency | Community cohort | US | ≥18 | SS ARI | 11 | 15 | 755.05 [376.92, 1262.34] |
| Milstone, 2006 | Seasonal | Immunodeficiency | Community cohort | US | ≥18 | SS ARI | 8 | 21 | 384 [165.78, 692.29] |
| Lopez-Medrano, 2007 | Seasonal | Immunodeficiency | Community cohort | ES | ≥18 | SS RVI | 6 | 89 | 67.67 [24.83, 131.6] |
| *Overall estimate for immunodeficient (seasonal) (Q=22.76, .98; p=0.00; $I^2$ = 88.0%)* | | | | | | | | | *260.89 [82.33,826.65]* |
| Falsey, 2005 | Seasonal | Cardiopulmonary | Community cohort | US | ≥21 | SS ARI | 56 | 900 | 62.26 [47.03, 79.59] |
| Hutchinson, 2007 | Seasonal | Cardiopulmonary | Community cohort | AU | ≥18 | SS AEC | 1 | 82 | 12.17 [0.31, 44.88] |
| Belongia, 2018* | Seasonal | Cardiopulmonary | Medically attended | US | ≥60 | SS ARI | 109 | 13017 | 8.37 [6.88, 10.02] |
| *Overall estimate for cardiopulmonary (seasonal) (Q=148.98, p=0.00; $I^2$ = 97.0%)* | | | | | | | | | *19.15 [6.06, 60.49]* |

*SS = systematic sampling, CI = sampling by clinical indication, 95% CI = 95% confidence interval, ARI = acute respiratory infection, AEC = acute exacerbation of COPD; RVI = respiratory virus infection. Belongia, 2018: Fever & cough included in eligibility criteria most seasons.*

**Supplementary Table 13.** RSV related healthcare utilisation of older and HR adults.

| Study | Country | Data collection | Population & age | Sampling | n | RSV+ | Total participants | Proportion [95% CI] |
|---|---|---|---|---|---|---|---|---|
| ***Healthcare utilisation in older adults*** | | | | | | | | |
| *Outpatient visits among RSV-positive older adults* | | | | | | | | |
| Falsey, 2005 | US | Seasonal | Community cohort, ≥65 | SS ARI | 8 | 46 | 608 | 17.39 [9.09, 30.72] |
| *Emergency department visits among RSV-positive older adults* | | | | | | | | |
| Belongia, 2018 | US | Seasonal | Medically attended, ≥65 | SS ARI | 13 | 243 | 2257 | 5.35 [3.15, 8.94] |
| Falsey, 2005 | US | Seasonal | Community cohort, ≥60 | SS ARI | 0 | 46 | 608 | 0.00 [0.00, 7.71] |
| *Discharge to care among RSV-positive older adults* | | | | | | | | |
| Belongia, 2018 | US | Seasonal | Medically attended, ≥60 | SS ARI | 2 | 243 | 2257 | 0.82 [0.23, 2.95] |
| *Oxygen use among RSV-positive older adults* | | | | | | | | |
| Aronen, 2019 | FI | Annual | IP ≥65 | SS ARI | 3 | 22 | 382 | 13.64 [4.75, 33.33] |
| Belongia, 2018 | US | Seasonal | Medically attended, ≥60 | SS ARI | 36 | 243 | 2257 | 14.81 [10.90, 19.83] |
| *Antibiotic use among RSV-positive older adults* | | | | | | | | |
| Falsey, 2005 | US | Seasonal | Community cohort, ≥65 | SS ARI | 67 | 86 | 86 | 77.91 [68.05, 85.38] |
| Belongia, 2018 | US | Seasonal | Medically attended, ≥60 | SS ARI | 187 | 243 | 2257 | 76.95 [71.27, 81.81] |
| ***Healthcare utilisation in HR adults*** | | | | | | | | |
| *Outpatient visits among RSV-positive HR adults* | | | | | | | | |
| ***Cardiopulmonary*** | | | | | | | | |
| Falsey, 2005 | US | Seasonal | Community cohort, ≥21 | SS ARI | 16 | 56 | 540 | 28.57 [18.42, 41.48] |
| *Emergency department visits among RSV-positive HR adults* | | | | | | | | |
| ***Cardiopulmonary*** | | | | | | | | |
| Falsey, 2005 | US | Seasonal | Community cohort, ≥21 | SS ARI | 5 | 56 | 540 | 8.93 [3.87, 19.26] |
| *Discharge to care among RSV-positive HR adults* | | | | | | | | |
| ***Chronic Kidney Disease*** | | | | | | | | |
| Schmidt, 2019 | US | Annual | RSV+ IP ≥18 | CI | 12 | 107 | 489 | 11.21 [6.53, 18.59] |
| ***Diabetes*** | | | | | | | | |
| Schmidt, 2019 | US | Annual | RSV+ IP ≥18 | CI | 23 | 133 | 489 | 17.29 [11.81, 24.61] |
| ***Immunodeficient*** | | | | | | | | |
| Schmidt, 2019 | US | Annual | RSV+ IP ≥18 | CI | 9 | 216 | 489 | 4.17 [2.21, 7.73] |
| *Oxygen use among RSV-positive HR adults* | | | | | | | | |
| ***Immunodeficiency*** | | | | | | | | |
| Li, 2012 | US | Annual | RSV+ ≥18 | CI | 5 | 21 | 21 | 23.81 [10.63, 45.09] |
| Gueller, 2013 | DE | Seasonal | IP ≥18 | SS | 5 | 10 | 29 | 50.00 [23.66, 76.34] |
| ***Institutionalized older adults*** | | | | | | | | |
| Hequet, 2019 | CH | Annual | Community cohort, ≥18 | SS ILI | 12 | 38 | 509 | 31.58 [19.08, 47.46] |

*IP = inpatients, SS = systematic sampling, CI = sampling by clinical indication, ILI = influenza like illness, ARI = acute respiratory infection*

.
